# BIAFLOWS: A Collaborative Framework to Reproducibly Deploy and Benchmark Bioimage Analysis Workflows

**DOI:** 10.1016/j.patter.2020.100040

**Published:** 2020-06-03

**Authors:** Ulysse Rubens, Romain Mormont, Lassi Paavolainen, Volker Bäcker, Benjamin Pavie, Leandro A. Scholz, Gino Michiels, Martin Maška, Devrim Ünay, Graeme Ball, Renaud Hoyoux, Rémy Vandaele, Ofra Golani, Stefan G. Stanciu, Natasa Sladoje, Perrine Paul-Gilloteaux, Raphaël Marée, Sébastien Tosi

**Affiliations:** 1 Montefiore Institute, University of Liège, 4000 Liège, Belgium; 2 FIMM, HiLIFE, University of Helsinki, 00014 Helsinki, Finland; 3 MRI, BioCampus Montpellier, Montpellier 34094, France; 4 VIB BioImaging Core, 3000 Leuven, Belgium; 5 Universidade Federal do Paraná, Curitiba 80060-000, Brazil; 6 HEPL, University of Liège, 4000 Liège, Belgium; 7 Masaryk University, 601 77 Brno, Czech Republic; 8 Faculty of Engineering İzmir, Demokrasi University, 35330 Balçova, Turkey; 9 Dundee Imaging Facility, School of Life Sciences, University of Dundee, Dundee DD1 5EH, UK; 10 Cytomine SCRL FS, 4000 Liège, Belgium; 11 Life Sciences Core Facilities, Weizmann Institute of Science, Rehovot 7610001, Israel; 12 Politehnica Bucarest, Bucarest 060042, Romania; 13 Uppsala University, P.O. Box 256, 751 05 Uppsala, Sweden; 14 Structure Fédérative de Recherche François Bonamy, Université de Nantes, CNRS, INSERM, Nantes Cedex 1 13522 44035, France; 15 Institute for Research in Biomedicine, IRB Barcelona, Barcelona Institute of Science and Technology, BIST, 08028 Barcelona, Spain

**Keywords:** image analysis, software, benchmarking, deployment, reproducibility, web application, community, bioimaging, deep learning

## Abstract

Image analysis is key to extracting quantitative information from scientific microscopy images, but the methods involved are now often so refined that they can no longer be unambiguously described by written protocols. We introduce BIAFLOWS, an open-source web tool enabling to reproducibly deploy and benchmark bioimage analysis workflows coming from any software ecosystem. A curated instance of BIAFLOWS populated with 34 image analysis workflows and 15 microscopy image datasets recapitulating common bioimage analysis problems is available online. The workflows can be launched and assessed remotely by comparing their performance visually and according to standard benchmark metrics. We illustrated these features by comparing seven nuclei segmentation workflows, including deep-learning methods. BIAFLOWS enables to benchmark and share bioimage analysis workflows, hence safeguarding research results and promoting high-quality standards in image analysis. The platform is thoroughly documented and ready to gather annotated microscopy datasets and workflows contributed by the bioimaging community.

## Highlights

• Image analysis is inescapable in extracting quantitative data from scientific images
• It can be difficult to deploy and apply state-of-the-art image analysis methods
• Comparing heterogeneous image analysis methods is tedious and error prone
• We introduce a platform to deploy and fairly compare image analysis workflows

## The Bigger Picture

Image analysis is currently one of the major hurdles in the bioimaging chain, especially for large datasets. BIAFLOWS seeds the ground for virtual access to image analysis workflows running in high-performance computing environments. Providing a broader access to state-of-the-art image analysis is expected to have a strong impact on research in biology, and in other fields where image analysis is a critical step in extracting scientific results from images. BIAFLOWS could also be adopted as a federated platform to publish microscopy images together with the workflows that were used to extract scientific data from these images. This is a milestone of open science that will help to accelerate scientific progress by fostering collaborative practices.

## Teaser

While image analysis is becoming inescapable in the extraction of quantitative information from scientific images, it is currently challenging for life scientists to find, test, and compare state-of-the-art image analysis methods compatible with their own microscopy images. It is also difficult and time consuming for algorithm developers to validate and reproducibly share their methods. BIAFLOWS is a web platform addressing these needs. It can be used as a local solution or through an immediately accessible and curated online instance.

## Introduction

As life scientists collect microscopy datasets of increasing size and complexity,^1^ computational methods to extract quantitative information from these images have become inescapable. In turn, modern image analysis methods are becoming so complex (often involving a combination of image-processing steps and deep-learning methods) that they require expert configuration to run. Unfortunately, the software implementations of these methods are commonly shared as poorly reusable and scarcely documented source code and seldom as user-friendly packages for mainstream bioimage analysis (BIA) platforms.^2^, ^3^, ^4^ Even worse, test images are not consistently provided with the software, and it can hence be difficult to identify the baseline for valid results or the critical adjustable parameters to optimize the analysis. Altogether, this does not only impair the reusability of the methods and impede reproducing published results^5^^,^^6^ but also makes it difficult to adapt these methods to process similar images. To improve this situation, scientific datasets are now increasingly made available through public web-based applications^7^, ^8^, ^9^ and open-data initiatives,^10^ but existing platforms do not systematically offer advanced features such as the ability to view and process multidimensional images online or to let users assess the quality of the analysis against a ground-truth reference (also known as benchmarking). Benchmarking is at the core of biomedical image analysis challenges and it a practice known to sustain the continuous improvement of image analysis methods and promote their wider diffusion.^11^ Unfortunately, challenges are rather isolated competitions and they suffer from known limitations^12^: each event focuses on a single image analysis problem, and it relies on ad hoc data formats and scripts to compute benchmark metrics. Both challenge organizers and participants are therefore duplicating efforts from challenge to challenge, whereas participants' workflows are rarely available in a sustainable and reproducible fashion. Additionally, the vast majority of challenge datasets come from medical imaging, not from biology: for instance, as of January 2020, only 15 out of 198 datasets indexed in Grand Challenge^13^ were collected from fluorescence microscopy, one of the most common imaging modalities for research in biology. As a consequence, efficient BIA methods are nowadays available but their reproducible deployment and benchmarking are still stumbling blocks for open science. In practice, end users are faced with a plethora of BIA ecosystems and workflows to choose from, and they have a hard time reproducing results, validating their own analysis, or ensuring that a given method is the most appropriate for the problem they face. Likewise, developers cannot systematically validate the performance of their BIA workflows on public datasets or compare their results to previous work without investing time-consuming and error-prone reimplementation efforts. Finally, it is challenging to make BIA workflows available to the whole scientific community in a configuration-free and reproducible manner.

## Results

### Conception of Software Architecture for Reproducible Deployment and Benchmarking

**Figure 1:**
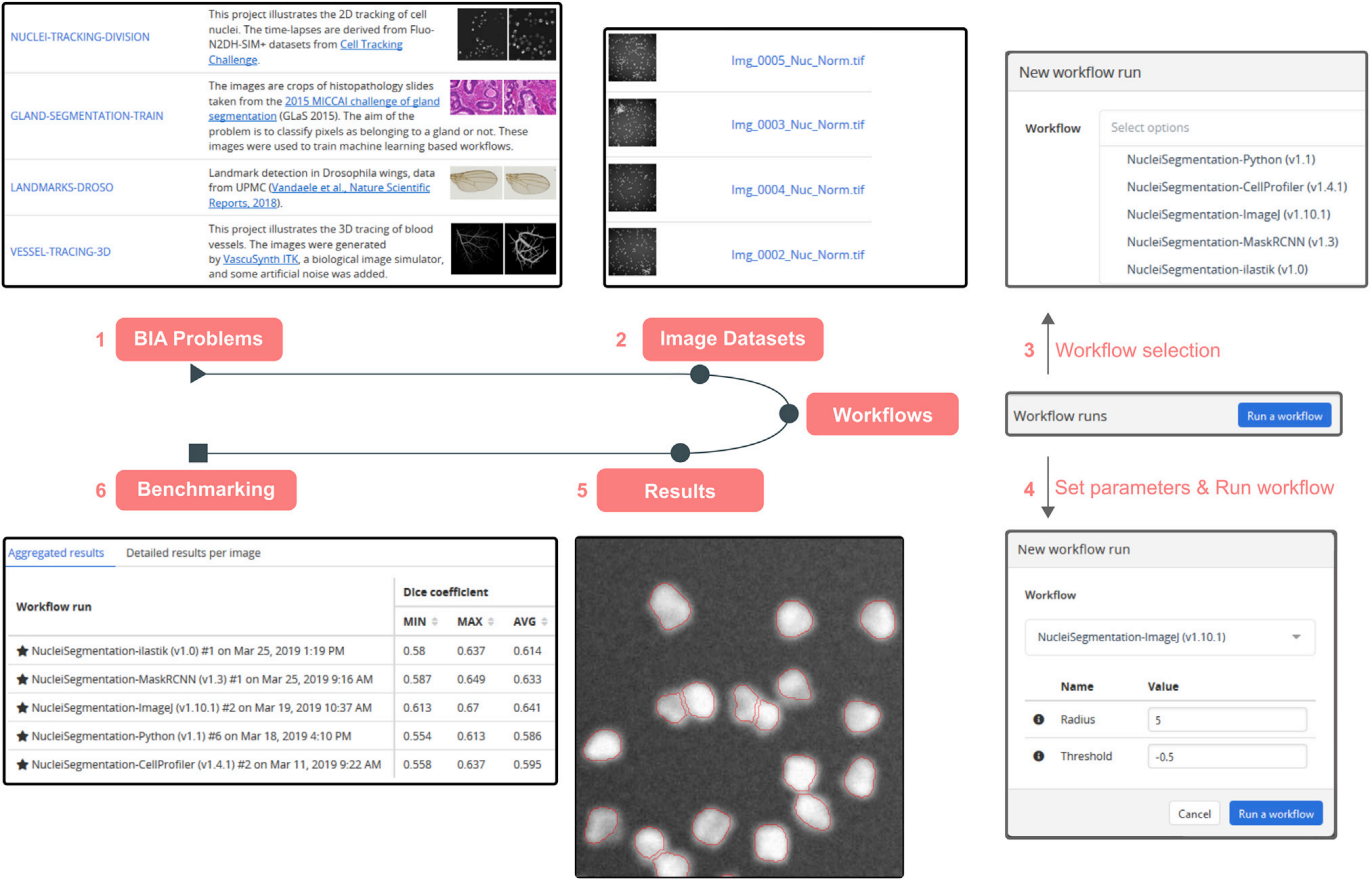
BIAFLOWS Web Interface (1) Users select a BIA problem (Table S1) and (2) browse the images illustrating this problem, for instance to compare them with their own images, then (3) select a workflow (Table S1) and associated parameters (4) to process the images. The results can then be overlaid on the original images from the online image viewer (5), and (6) benchmark metrics can be browsed, sorted, and filtered both as overall statistics or per image.

Within the Network of European Bioimage Analysts (NEUBIAS COST [www.cost.eu] Action CA15124), an important body of work focuses on channeling the efforts of bioimaging stakeholders (including biologists, bioimage analysts, and software developers) to ensure a better characterization of existing bioimage analysis workflows and to bring these tools to a larger number of scientists. Together, we have envisioned and implemented BIAFLOWS (Figure 1), a community-driven, open-source web platform to reproducibly deploy and benchmark bioimage analysis workflows on annotated multidimensional microscopy data. Whereas some emerging bioinformatics web platforms^14^^,^^15^ simply rely on “Dockerized” (https://www.docker.com/resources/what-container) environments and interactive Python notebooks to access and process scientific data from public repositories, BIAFLOWS offers a versatile and extensible integrated framework to (1) import annotated image datasets and organize them into BIA problems, (2) encapsulate BIA workflows regardless of their target software, (3) batch process the images, (4) remotely visualize the images together with the results, and (5) automatically assess the performance of the workflows from widely accepted benchmark metrics.

**Figure 2:**
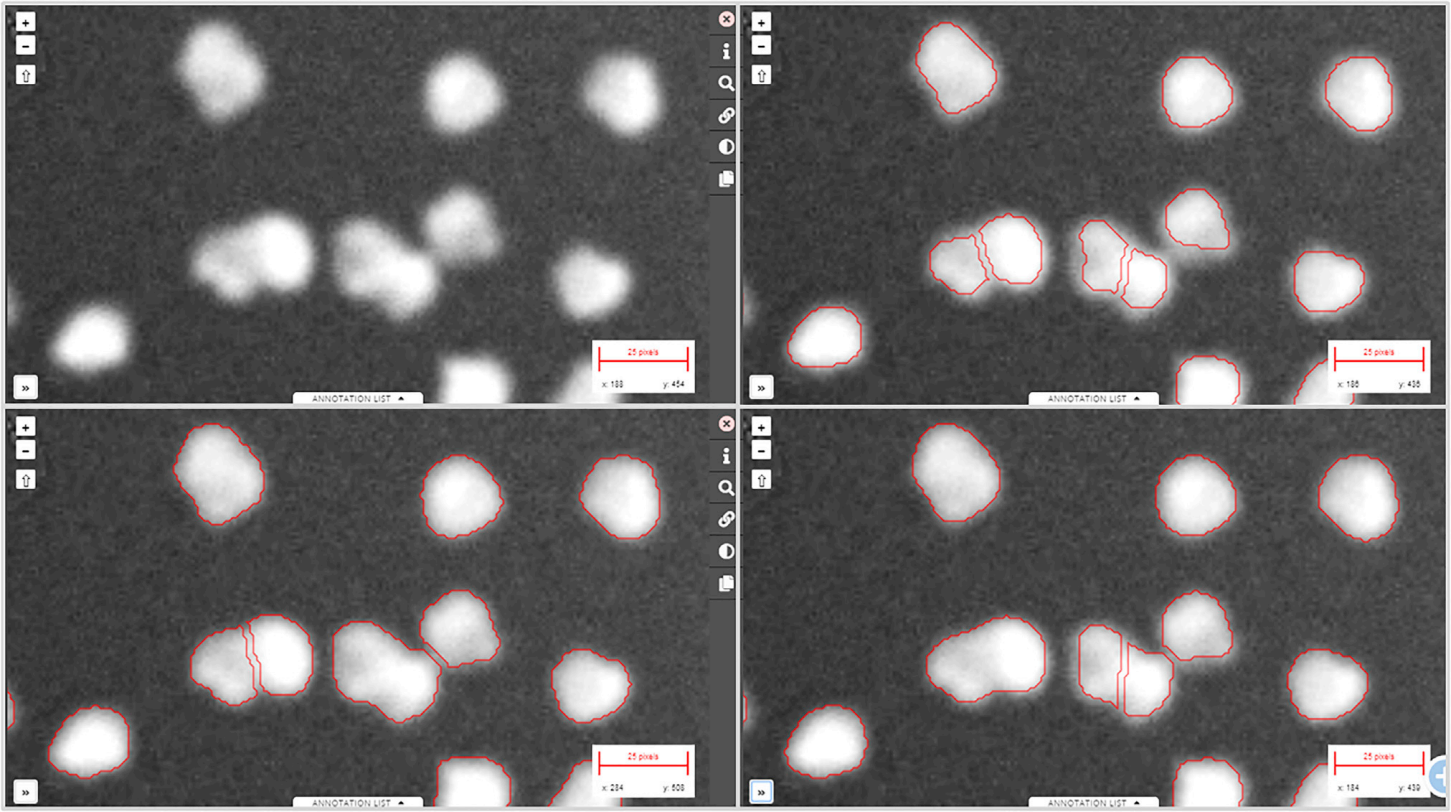
Synchronizing Image Viewers Displaying Different Workflow Results Region from one of the sample images available in NUCLEI-SEGMENTATION problem (accessible from the BIAFLOWS online instance). Original image (upper left), same image overlaid with results from: custom ImageJ macro (upper right), custom CellProfiler pipeline (lower left), and custom Python script (lower right).

**Figure 3:**
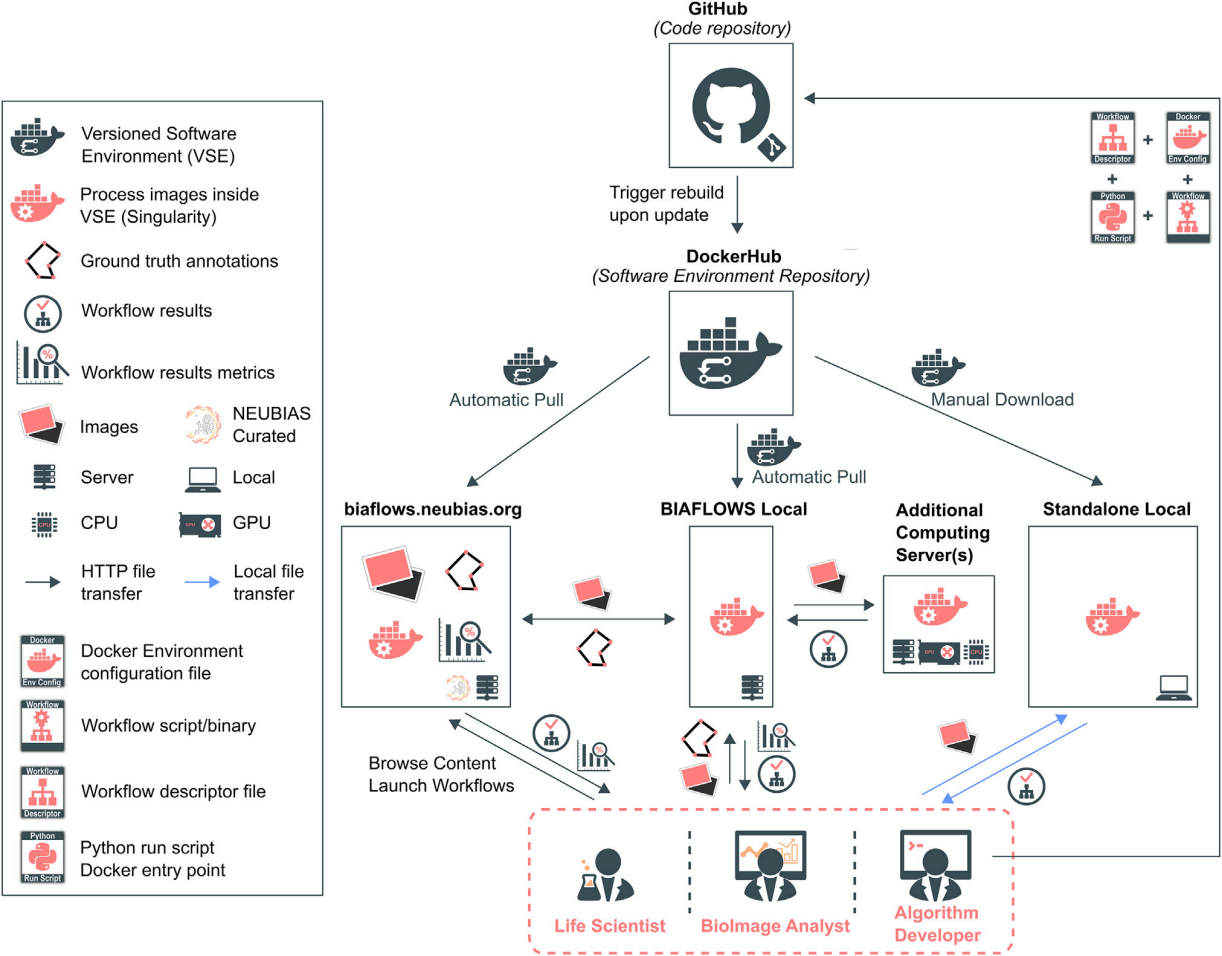
BIAFLOWS Architecture and Possible Deployment Scenarios Workflows are hosted in a trusted source code repository (GitHub). Workflow (Docker) images encapsulate workflows together with their execution environments to ensure reproducibility. Workflow images are automatically built by a cloud service (DockerHub) whenever a new workflow is released or an existing workflow is updated from its trusted GitHub repository. Different BIAFLOWS instances monitor DockerHub and pull new or updated workflow images, which can also be downloaded to process local images without BIAFLOWS (Standalone Local).

BIAFLOWS content can be interactively explored and triggered (Box 1) from a streamlined web interface (Figure 1). For a given problem, a set of standard benchmark metrics (Supplemental Experimental Procedures section 6) are reported for every workflow run, with accompanying technical and interpretation information available from the interface. One main metric is also highlighted as the most significant metric to globally rank the performance of the workflows. To complement benchmark results, workflow outputs can also be visualized simultaneously from multiple annotation layers or synchronized image viewers (Figure 2). BIAFLOWS is open-source and thoroughly documented (https://biaflows-doc.neubias.org/), and extends Cytomine,^16^ a web platform originally developed for the collaborative annotation of high-resolution bright-field bioimages. BIAFLOWS required extensive software development and content integration to enable the benchmarking of BIA workflows; accordingly, the web user interface has been completely redesigned to streamline this process (Figure 1). First, a module to upload multidimensional (C, Z, T) microscopy datasets and a fully fledged remote image viewer were implemented. Next, the architecture was refactored to enable the reproducible remote execution of BIA workflows encapsulated with their original software environment in Docker images (workflow images). To abstract out the operations performed by a workflow, we adopted a rich application description schema^17^ describing its interface (input, output, parameters) and default parameter values (Supplemental Experimental Procedures section 3). The system was also engineered to monitor trusted user spaces hosting a collection of workflow images and to automatically pull new or updated workflows (Figure 3, DockerHub). In turn, workflow images are built and versioned in the cloud whenever a new release is triggered from their associated source code repositories (Figure 3, GitHub). To ensure reproducibility, we enforced that all versions of the workflow images are permanently stored and accessible from the system. Importantly, the workflows can be run on any computational resource, including high-performance computing and multiple server architectures. This is achieved by seamlessly converting the workflow images to a compatible format (Singularity^18^), and dispatching them to the target computational resources over the network by SLURM^19^ (Figure 3, additional computing servers). To enable interoperability between all components, some standard object annotation formats were specified for important classes of BIA problems (Supplemental Experimental Procedures section 4). We also developed a software library to compute benchmark metrics associated with these problem classes by adapting and integrating the code from existing biomedical challenges^13^ and scientific publications.^20^ With this new design, benchmark metrics are automatically computed after every workflow run. BIAFLOWS can also be deployed on a local server to manage private images and workflows and to process images locally (Figure 3, BIAFLOWS local; Supplemental Experimental Procedures section 2). To simplify the coexistence of these different deployment scenarios, we developed migration tools (Supplementary Experimental Procedures section 5) to transfer content between existing BIAFLOWS instances (including the online instance described hereafter). Importantly, all content from any instance can be accessed programmatically through a RESTful interface, which ensures complete data accessibility and interoperability. Finally, for full flexibility, workflows can be downloaded manually from DockerHub to process local images independently of BIAFLOWS (Figure 3, standalone local; Supplemental Experimental Procedures section 5).

Box 1 **How to Get Started with BIAFLOWS**

• Watch BIAFLOWS video tutorial (https://biaflows.neubias.org).
• Visit BIAFLOWS documentation portal (https://biaflows-doc.neubias.org).
• Access BIAFLOWS online instance (https://biaflows.neubias.org) in read-only mode.This public instance is curated by NEUBIAS (http://neubias.org) and backed by bioimage analysts and software developers across the world. You can also access BIAFLOWS sandbox server (https://biaflows-sandbox.neubias.org/) without access restriction.
• Install your own BIAFLOWS instance on a desktop computer or a server to manage images locally or process them with existing BIAFLOWS workflows. Follow “Installing and populating BIAFLOWS locally” from the documentation portal.
• Download a workflow to process your own images locally. Follow “Executing a BIAFLOWS workflow without BIAFLOWS server” from the documentation portal.
• Share your thoughts and get help on our forum (https://forum.image.sc/tags/biaflows), or write directly to our developer team at biaflows@neubias.org.

### BIAFLOWS Online Curated Instance for Public Benchmarking

**Figure 4:**
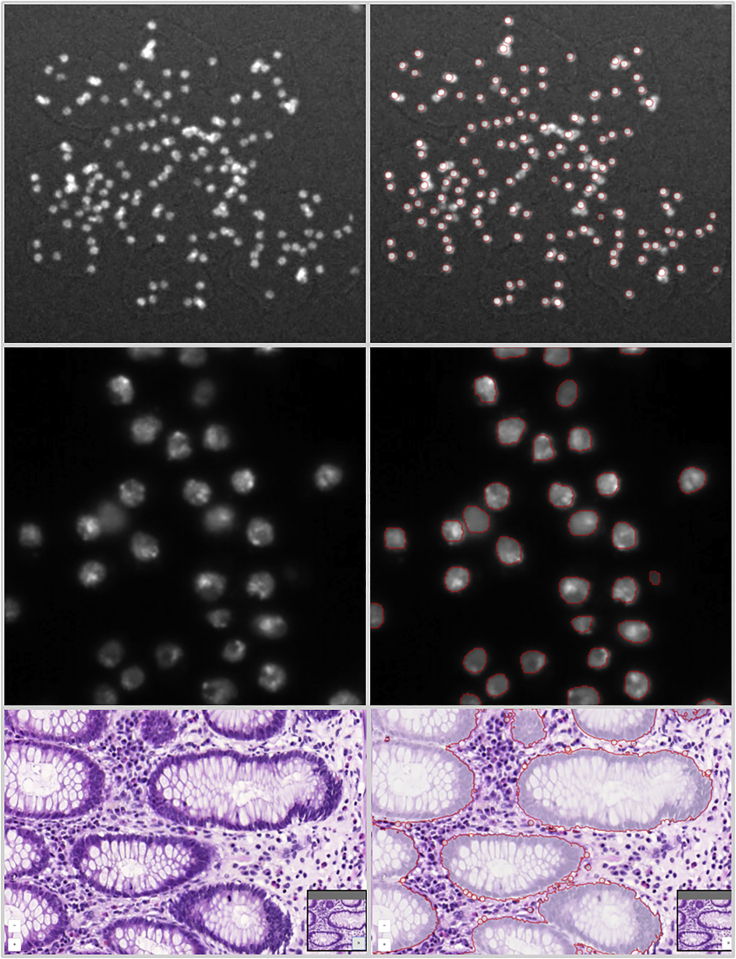
Sample Images from the BIAFLOWS Online Instance Illustrating Several BIA Problem Classes, and Results from Associated Workflows Original image (left) and workflow results (right), from top to bottom: (1) spot detection in synthetic images (SIMCEP^29^); (2) nuclei segmentation in images from Kaggle Data Science Bowl 2018;^24^ (3) pixel classification in images from 2015 MICCAI gland segmentation challenge.^40^

**Figure 5:**
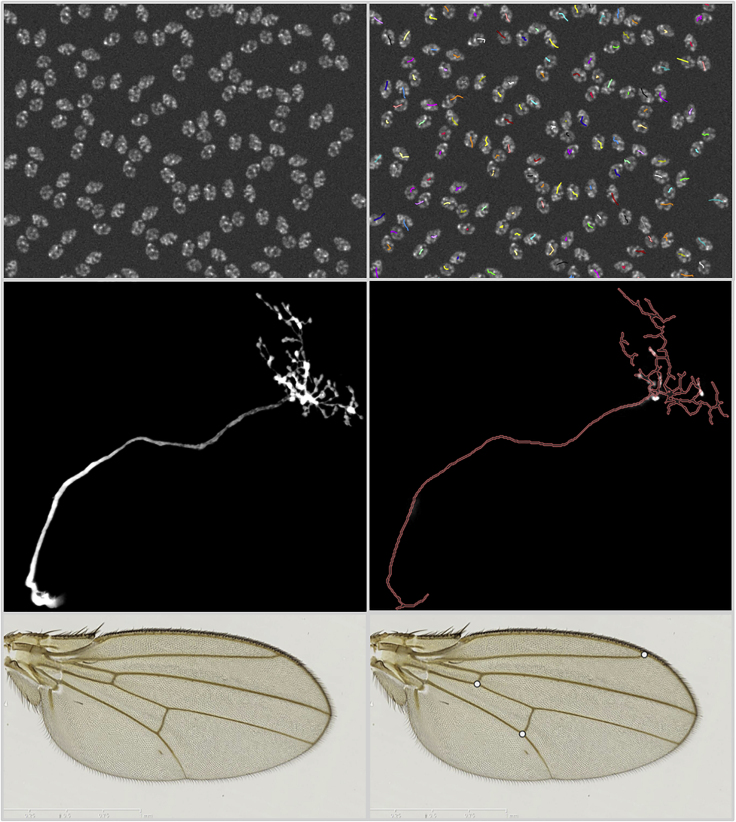
Sample Images from the BIAFLOWS Online Instance Illustrating Several BIA Problem Classes, and Results from Associated Workflows Original image (left) and workflow results (right), from top to bottom: (1) particle tracking in synthetic time-lapse displaying non-dividing nuclei (CytoPACQ^26^), single frame + dragon-tail tracks; (2) neuron tree tracing in 3D image stacks from DIADEM challenge,^21^ average intensity projection (left), traced skeleton z projection (dilated, red); (3) landmark detection in *Drosophila* wing images.^30^

An online instance of BIAFLOWS is maintained by NEUBIAS and available at https://biaflows.neubias.org/ (Figure 3). This server is ready to host community contributions and is already populated with a substantial collection of annotated image datasets illustrating common BIA problems and several associated workflows to process these images (Table S1). Concretely, we integrated BIA workflows spanning nine important BIA problem classes illustrated by 15 image datasets imported from existing challenges (DIADEM,^21^ Cell Tracking Challenge,^22^ Particle Tracking Challenge,^23^ Kaggle Data Science Bowl 2018^24^), created from synthetic data generators^25^ (CytoPacq,^26^ TREES toolbox,^27^ Vascusynth,^28^ SIMCEP^29^), or contributed by NEUBIAS members.^30^ The following problem classes are currently represented: object detection/counting, object segmentation, and pixel classification (Figure 4); particle tracking, object tracking, filament network tracing, filament tree tracing, and landmark detection (Figure 5). To demonstrate the versatility of the platform we integrated 34 workflows, each targeting a specific software or programming language: ImageJ/FIJI macros and scripts,^31^ Icy protocols,^32^ CellProfiler pipelines,^33^ Vaa3D plugins,^34^ ilastik pipelines,^35^ Octave scripts,^36^ Jupyter notebooks,^15^ and Python scripts leveraging Scikit-learn^37^ for supervised learning algorithms, and Keras^38^ or PyTorch^39^ for deep learning. This list, although already extensive, is not limited, as BIAFLOWS core architecture enables one to seamlessly add other software as long as they fulfill minimal requirements (Supplemental Experimental Procedures section 3). To demonstrate the potential of the platform to perform open benchmarking, a case study has been performed with (and is available from) BIAFLOWS to compare workflows identifying nuclei in microscopy images. The content from the BIAFLOWS online instance (https://biaflows.neubias.org) can be viewed in read-only mode from the guest account, while the workflows can be launched from the sandbox server (https://biaflows-sandbox.neubias.org/). An extensive user guide and video tutorial are available online from the same URLs. To enhance their visibility, all workflows hosted in the system are also referenced from NEUBIAS Bioimage Informatics Search Index (http://biii.eu/). BIAFLOWS online instance is fully extensible and, with minimal effort, interested developers can package their own workflows (Supplemental Experimental Procedures section 3) and make them available for benchmarking (Box 2). Similarly, following our guidelines (Supplemental Experimental Procedures section 2), scientists can make their images and ground-truth annotations available online through the online instance or through a local instance they manage (Box 2). Finally, all online content can be seamlessly migrated to a local BIAFLOWS instance (Supplemental Experimental Procedures section 5) for further development or to process local images.

To further increase the content currently available in BIAFLOWS online instance, calls for contribution will be shortly launched to gather more annotated microscopy images and encourage developers to package their own workflows. The support of new problem classes is also planned, for example, to benchmark the detection of blinking events in the context of super-resolution localization microscopy or the detection of landmark points for image registration. There is no limitation in using BIAFLOWS in other fields where image analysis is a critical step in extracting scientific results from images, for instance material or plant science and biomedical imaging.

### Case Study: Comparing the Performance of Nuclei Segmentation by Classical Image Processing, Classical Machine Learning, and Deep-Learning Methods

To illustrate how to use BIAFLOWS for the open benchmarking of BIA workflows, we integrated seven nuclei segmentation workflows (Supplemental Experimental Procedures section 1). All content (images, ground-truth annotations, workflows, benchmark results) is readily accessible from the BIAFLOWS online instance. The workflows were benchmarked on two different image datasets: a synthetic dataset of ten images generated^29^ for the purpose of this study, and a subset of 65 images from an existing nuclei segmentation challenge (Kaggle Data Science Bowl 2018^24^). The study was articulated in three parts: (1) evaluating the performance of three BIA workflows implementing classical methods to identify nuclei (synthetic dataset); (2) evaluating the performance of three ubiquitous deep-learning workflows on the same dataset; and (3) evaluating the performance of these deep-learning workflows (and a classical machine-learning workflow) on Kaggle Data Science Bowl 2018 (KDSB2018) subset. As a baseline, the classical workflows were manually tuned to obtain the best performance on the synthetic dataset while the machine-learning workflows were trained on generic nuclei image datasets with no further tuning for the synthetic dataset. Despite this, the deep-learning methods proved to be almost as accurate, or in some cases more accurate, than the best classical method (Tables S2 and S3). It was also evidenced that a set of benchmark metrics is generally to be favored over a single metric, since some widely used metrics only capture a single aspect of a complex problem. For instance, object segmentation does not only aim at accurately discriminating foreground from background pixels (assessed by DICE-like metrics) but overall at identifying independent objects (for instance to further measure their geometrical properties). Also, the visual inspection of workflow results proved useful in understanding the underlying errors evidenced by poor benchmark metrics results (Figure S1). All these features are readily available in BIAFLOWS, which swiftly enables to link workflow source code, benchmark metrics results, and visual results. The same methodology can be easily translated to other experiments.

## Discussion

BIAFLOWS addresses a number of critical requirements to foster open image analysis for life sciences: (1) sharing and visualizing annotated microscopy images illustrating commonly faced BIA problems; (2) sharing reproducible BIA workflows; (3) exposing workflow parameters and associated default values; (4) computing relevant benchmark metrics to compare workflows performance; and (5) providing a standard way to store, visualize, and share BIA workflows results. As such, BIAFLOWS is a central asset for biologists and bioimage analysts to leverage state-of-the-art bioimaging methods and efficiently reuse them in a different context. It is also a tool of choice for algorithm developers and challenge organizers to benchmark bioimage analysis workflows. Challenge participants traditionally reported workflow predictions on websites such as Kaggle and grand-challenge.org. The latter is currently developing a Docker-based mechanism (https://grand-challengeorg.readthedocs.io/en/latest/evaluation.html#) to package workflows (mostly coming from medical imaging), but these platforms do not offer a complete integrated web environment to host image datasets, automatically import workflows from open-source repositories, automate benchmark metric computation, and remotely visualize all results in a streamlined web interface such as BIAFLOWS. We believe BIAFLOWS could be made interoperable with the grand-challenge.org Docker-based mechanism to package workflows, and used by challenge organizers as a fully integrated platform to automate benchmarking and share challenge results in a more reproducible way. Finally, BIAFLOWS provides a solution to authors willing to share online supporting data, methods, and results associated with their published scientific results.

With respect to sustainability and scalability, BIAFLOWS is backed by a team of senior bioimage analysts and software developers. The software is compatible with high-performance computing environments and is based on Cytomine architecture,^16^ which has already proved itself capable of serving large datasets to many users simultaneously.^41^ We invested a large amount of effort in documenting BIAFLOWS, and the online instance is ready to receive hundreds of new image datasets and workflows as community contributions (Box 2). To increase the content of BIAFLOWS online instance, we will briefly launch calls for contributions targeting existing BIAFLOWS problem classes. We propose that BIAFLOWS becomes a hub for BIA methods developers, bioimage analysts, and life scientists to share annotated datasets, reproducible BIA workflows, and associated results from benchmark and research studies. In future work, we will work toward interoperability with existing European image storage and workflow management infrastructures such as BioImage Archive,^42^ https://www.eosc-life.eu/, and Galaxy,^15^ and further improve the scalability and sustainability of the platform.

Box 2 **How to Contribute to BIAFLOWS**

• Scientists can contribute published annotated microscopy images to BIAFLOWS online instance. See “Problem classes, ground truth annotations and reported metrics” from the documentation portal for information on the expected images and ground-truth annotations formats, and contact us through the dedicated thread on https://forum.image.sc/tags/biaflows.
• To showcase a workflow in the BIAFLOWS online instance, developers can encapsulate their source code, test it on a local BIAFLOWS instance or BIAFLOWS sandbox server (https://biaflows-sandbox.neubias.org/), and open an issue in this GitHub repository: https://github.com/Neubias-WG5/SubmitToBiaflows. Follow “Creating a BIA workflow and adding it to a BIAFLOWS instance” from the documentation portal.
• Feature requests or bug reports can be posted to BIAFLOWS GitHub (https://github.com/neubias-wg5).
• Users can contribute to the documentation by submitting a pull request to https://github.com/Neubias-WG5/neubias-wg5.github.io.
• Any user can share data and results, e.g., accompanying scientific publications, via “Access BIAFLOWS from a Jupyter notebook” from the documentation portal or by directly linking the content of a BIAFLOWS instance.

## Experimental Procedures

### Resource Availability

#### Lead Contact

Further information and requests for resources should be directed to the Lead Contact, Sébastien Tosi (sebastien.tosi@irbbarcelona.org).

#### Materials Availability

No materials were used in this study.

#### Data and Code Availability

BIAFLOWS is an open-source project and its source code can be freely downloaded at https://github.com/Neubias-WG5.

All images and annotations described and used in this article can be downloaded from the BIAFLOWS online instance at https://biaflows.neubias.org/.

A sandbox server from which all workflows available in BIAFLOWS online instance can be launched remotely, and new workflows/datasets appended for testing are available at https://biaflows-sandbox.neubias.org/.

The documentation to install, use, and extend the software is available at https://neubias-wg5.github.io/.

## Supplemental Information

Document S1. Supplemental Experimental Procedures, Figure S1, and Tables S1–S3

Document S2. Article plus Supplemental Information
