# Supplementary material for: BIAFLOWS: A Collaborative Framework to Reproducibly Deploy and Benchmark Bioimage Analysis Workflows
Source: Patterns (N Y). 2020 Jun 3;1(3):100040. doi: 10.1016/j.patter.2020.100040 (PMC7660398; doi:10.1016/j.patter.2020.100040)
Supplement: Document S2. Article plus Supplemental Information [file mmc2.pdf]

Descriptor

Patterns

BIAFLOWS: A Collaborative Framework to Reproducibly Deploy and Benchmark Bioimage Analysis Workflows

Highlights

- Image analysis is inescapable in extracting quantitative data from scientific images
- It can be difficult to deploy and apply state-of-the-art image analysis methods
- Comparing heterogeneous image analysis methods is tedious and error prone
- We introduce a platform to deploy and fairly compare image analysis workflows

Authors

Ulysse Rubens, Romain Mormont, Lassi Paavolainen, ..., Perrine Paul-Gilloteaux, Raphaël Marée, Sébastien Tosi

Correspondence

sebastien.tosi@irbbarcelona.org

In Brief

While image analysis is becoming inescapable in the extraction of quantitative information from scientific images, it is currently challenging for life scientists to find, test, and compare state-of-the-art image analysis methods compatible with their own microscopy images. It is also difficult and time consuming for algorithm developers to validate and reproducibly share their methods. BIAFLOWS is a web platform addressing these needs. It can be used as a local solution or through an immediately accessible and curated online instance.

Rubens et al., 2020, Patterns *1*, 100040
June 12, 2020 © 2020 The Author(s).
https://doi.org/10.1016/j.patter.2020.100040

CellPress

Patterns

CellPress
OPEN ACCESS

Descriptor

BIAFLOWS: A Collaborative Framework to Reproducibly Deploy and Benchmark Bioimage Analysis Workflows

Ulysse Rubens,[1,16] Romain Mormont,[1,16] Lassi Paavolainen,[2] Volker Bäcker,[3] Benjamin Pavie,[4] Leandro A. Scholz,[5] Gino Michiels,[6] Martin Maška,[7] Devrim Ünay,[8] Graeme Ball,[9] Renaud Hoyoux,[10] Rémy Vandaele,[1] Ofra Golani,[11] Stefan G. Stanciu,[12] Natasa Sladoje,[13] Perrine Paul-Gilloteaux,[14] Raphaël Marée,[1,17] and Sébastien Tosi[15,17,18,*]

[1]Montefiore Institute, University of Liège, 4000 Liège, Belgium
[2]FIMM, HiLIFE, University of Helsinki, 00014 Helsinki, Finland
[3]MRI, BioCampus Montpellier, Montpellier 34094, France
[4]VIB BioImaging Core, 3000 Leuven, Belgium
[5]Universidade Federal do Paraná, Curitiba 80060-000, Brazil
[6]HEPL, University of Liège, 4000 Liège, Belgium
[7]Masaryk University, 601 77 Brno, Czech Republic
[8]Faculty of Engineering İzmir, Demokrasi University, 35330 Balçova, Turkey
[9]Dundee Imaging Facility, School of Life Sciences, University of Dundee, Dundee DD1 5EH, UK
[10]Cytomine SCRL FS, 4000 Liège, Belgium
[11]Life Sciences Core Facilities, Weizmann Institute of Science, Rehovot 7610001, Israel
[12]Politehnica Bucarest, Bucarest 060042, Romania
[13]Uppsala University, P.O. Box 256, 751 05 Uppsala, Sweden
[14]Structure Fédérative de Recherche François Bonamy, Université de Nantes, CNRS, INSERM, Nantes Cedex 1 13522 44035, France
[15]Institute for Research in Biomedicine, IRB Barcelona, Barcelona Institute of Science and Technology, BIST, 08028 Barcelona, Spain
[16]These authors contributed equally
[17]These authors contributed equally
[18]Lead Contact
*Correspondence: sebastien.tosi@irbbarcelona.org
https://doi.org/10.1016/j.patter.2020.100040

THE BIGGER PICTURE Image analysis is currently one of the major hurdles in the bioimaging chain, especially for large datasets. BIAFLOWS seeds the ground for virtual access to image analysis workflows running in high-performance computing environments. Providing a broader access to state-of-the-art image analysis is expected to have a strong impact on research in biology, and in other fields where image analysis is a critical step in extracting scientific results from images. BIAFLOWS could also be adopted as a federated platform to publish microscopy images together with the workflows that were used to extract scientific data from these images. This is a milestone of open science that will help to accelerate scientific progress by fostering collaborative practices.

1 2 3 **4** 5 **Production:** Data science output is validated, understood, and regularly used for multiple domains/platforms

SUMMARY

Image analysis is key to extracting quantitative information from scientific microscopy images, but the methods involved are now often so refined that they can no longer be unambiguously described by written protocols. We introduce BIAFLOWS, an open-source web tool enabling to reproducibly deploy and benchmark bioimage analysis workflows coming from any software ecosystem. A curated instance of BIAFLOWS populated with 34 image analysis workflows and 15 microscopy image datasets recapitulating common bioimage analysis problems is available online. The workflows can be launched and assessed remotely by comparing their performance visually and according to standard benchmark metrics. We illustrated these features by comparing seven nuclei segmentation workflows, including deep-learning methods. BIAFLOWS enables to benchmark and share bioimage analysis workflows, hence safeguarding research results and

This is an open access article under the CC BY license (http://creativecommons.org/licenses/by/4.0/).

promoting high-quality standards in image analysis. The platform is thoroughly documented and ready to gather annotated microscopy datasets and workflows contributed by the bioimaging community.

INTRODUCTION

As life scientists collect microscopy datasets of increasing size and complexity,[1] computational methods to extract quantitative information from these images have become inescapable. In turn, modern image analysis methods are becoming so complex (often involving a combination of image-processing steps and deep-learning methods) that they require expert configuration to run. Unfortunately, the software implementations of these methods are commonly shared as poorly reusable and scarcely documented source code and seldom as user-friendly packages for mainstream bioimage analysis (BIA) platforms.[2–4] Even worse, test images are not consistently provided with the software, and it can hence be difficult to identify the baseline for valid results or the critical adjustable parameters to optimize the analysis. Altogether, this does not only impair the reusability of the methods and impede reproducing published results[5,6] but also makes it difficult to adapt these methods to process similar images. To improve this situation, scientific datasets are now increasingly made available through public web-based applications[7–9] and open-data initiatives,[10] but existing platforms do not systematically offer advanced features such as the ability to view and process multidimensional images online or to let users assess the quality of the analysis against a ground-truth reference (also known as benchmarking). Benchmarking is at the core of biomedical image analysis challenges and it a practice known to sustain the continuous improvement of image analysis methods and promote their wider diffusion.[11] Unfortunately, challenges are rather isolated competitions and they suffer from known limitations[12]: each event focuses on a single image analysis problem, and it relies on ad hoc data formats and scripts to compute benchmark metrics. Both challenge organizers and participants are therefore duplicating efforts from challenge to challenge, whereas participants' workflows are rarely available in a sustainable and reproducible fashion. Additionally, the vast majority of challenge datasets come from medical imaging, not from biology: for instance, as of January 2020, only 15 out of 198 datasets indexed in Grand Challenge[13] were collected from fluorescence microscopy, one of the most common imaging modalities for research in biology. As a consequence, efficient BIA methods are nowadays available but their reproducible deployment and benchmarking are still stumbling blocks for open science. In practice, end users are faced with a plethora of BIA ecosystems and workflows to choose from, and they have a hard time reproducing results, validating their own analysis, or ensuring that a given method is the most appropriate for the problem they face. Likewise, developers cannot systematically validate the performance of their BIA workflows on public datasets or compare their results to previous work without investing time-consuming and error-prone reimplementation efforts. Finally, it is challenging to make BIA workflows available to the whole scientific community in a configuration-free and reproducible manner.

RESULTS

Conception of Software Architecture for Reproducible Deployment and Benchmarking

Within the Network of European Bioimage Analysts (NEUBIAS COST [www.cost.eu] Action CA15124), an important body of work focuses on channeling the efforts of bioimaging stakeholders (including biologists, bioimage analysts, and software developers) to ensure a better characterization of existing bioimage analysis workflows and to bring these tools to a larger number of scientists. Together, we have envisioned and implemented BIAFLOWS (Figure 1), a community-driven, open-source web platform to reproducibly deploy and benchmark bioimage analysis workflows on annotated multidimensional microscopy data. Whereas some emerging bioinformatics web platforms[14,15] simply rely on "Dockerized" (https://www.docker.com/resources/what-container) environments and interactive Python notebooks to access and process scientific data from public repositories, BIAFLOWS offers a versatile and extensible integrated framework to (1) import annotated image datasets and organize them into BIA problems, (2) encapsulate BIA workflows regardless of their target software, (3) batch process the images, (4) remotely visualize the images together with the results, and (5) automatically assess the performance of the workflows from widely accepted benchmark metrics.

BIAFLOWS content can be interactively explored and triggered (Box 1) from a streamlined web interface (Figure 1). For a given problem, a set of standard benchmark metrics (Supplemental Experimental Procedures section 6) are reported for every workflow run, with accompanying technical and interpretation information available from the interface. One main metric is also highlighted as the most significant metric to globally rank the performance of the workflows. To complement benchmark results, workflow outputs can also be visualized simultaneously from multiple annotation layers or synchronized image viewers (Figure 2). BIAFLOWS is open-source and thoroughly documented (https://biaflows-doc.neubias.org/), and extends Cytomine,[16] a web platform originally developed for the collaborative annotation of high-resolution bright-field bioimages. BIAFLOWS required extensive software development and content integration to enable the benchmarking of BIA workflows; accordingly, the web user interface has been completely redesigned to streamline this process (Figure 1). First, a module to upload multidimensional (C, Z, T) microscopy datasets and a fully fledged remote image viewer were implemented. Next, the architecture was refactored to enable the reproducible remote execution of BIA workflows encapsulated with their original software environment in Docker images (workflow images). To abstract out the operations performed by a workflow, we adopted a rich application description schema[17] describing its interface (input, output, parameters) and default parameter values (Supplemental Experimental Procedures section 3). The system was also engineered to monitor trusted user spaces hosting a collection of workflow images and to automatically pull new or

CellPress
OPEN ACCESS

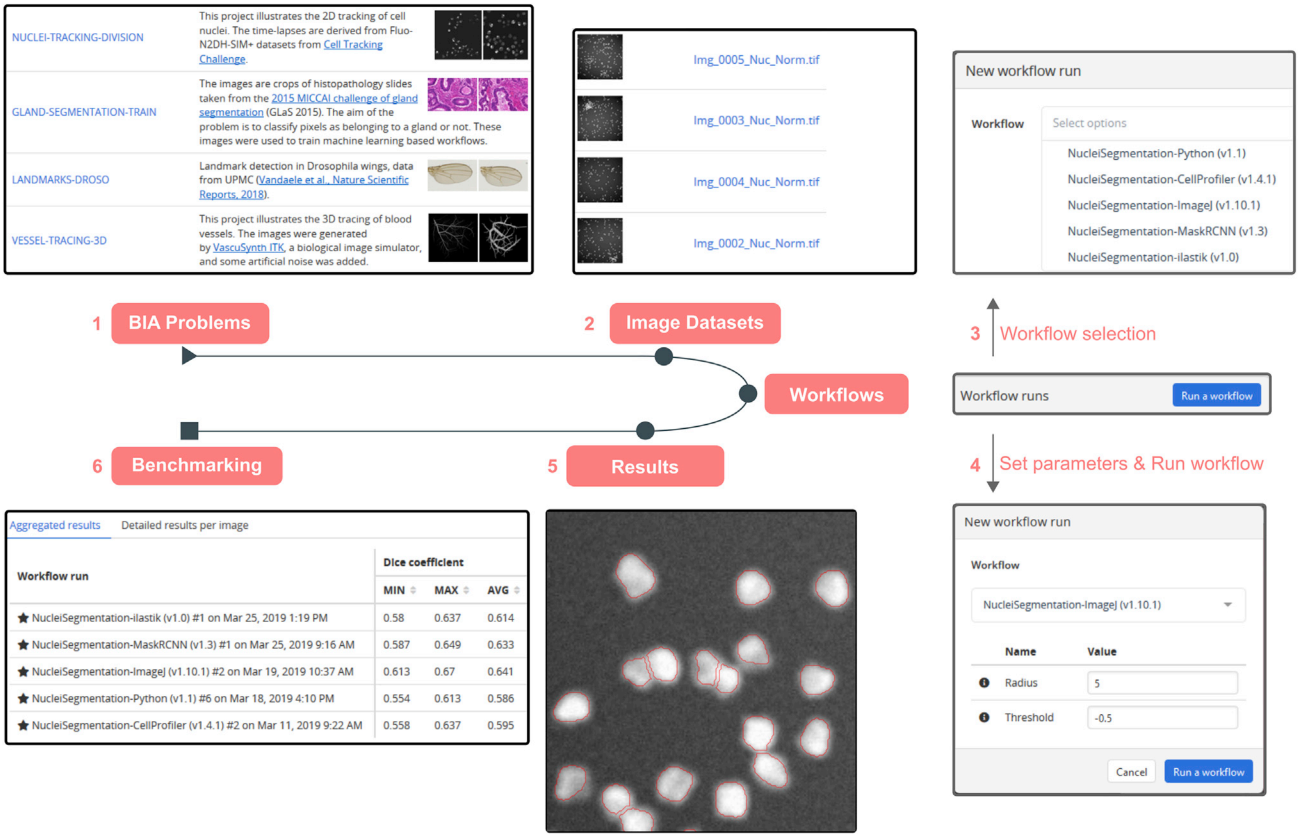

Figure 1. BIAFLOWS Web Interface

(1) Users select a BIA problem (Table S1) and (2) browse the images illustrating this problem, for instance to compare them with their own images, then (3) select a workflow (Table S1) and associated parameters (4) to process the images. The results can then be overlaid on the original images from the online image viewer (5), and (6) benchmark metrics can be browsed, sorted, and filtered both as overall statistics or per image.

updated workflows (Figure 3, DockerHub). In turn, workflow images are built and versioned in the cloud whenever a new release is triggered from their associated source code repositories (Figure 3, GitHub). To ensure reproducibility, we enforced that all versions of the workflow images are permanently stored and accessible from the system. Importantly, the workflows can be run on any computational resource, including high-performance computing and multiple server architectures. This is achieved by seamlessly converting the workflow images to a compatible format (Singularity[18]), and dispatching them to the target computational resources over the network by SLURM[19] (Figure 3, additional computing servers). To enable interoperability between all components, some standard object annotation formats were specified for important classes of BIA problems (Supplemental Experimental Procedures section 4). We also developed a software library to compute benchmark metrics associated with these problem classes by adapting and integrating the code from existing biomedical challenges[13] and scientific

Box 1. How to Get Started with BIAFLOWS

- Watch BIAFLOWS video tutorial (https://biaflows.neubias.org).
- Visit BIAFLOWS documentation portal (https://biaflows-doc.neubias.org).
- Access BIAFLOWS online instance (https://biaflows.neubias.org) in read-only mode.This public instance is curated by NEUBIAS (http://neubias.org) and backed by bioimage analysts and software developers across the world. You can also access BIAFLOWS sandbox server (https://biaflows-sandbox.neubias.org/) without access restriction.
- Install your own BIAFLOWS instance on a desktop computer or a server to manage images locally or process them with existing BIAFLOWS workflows. Follow "Installing and populating BIAFLOWS locally" from the documentation portal.
- Download a workflow to process your own images locally. Follow "Executing a BIAFLOWS workflow without BIAFLOWS server" from the documentation portal.
- Share your thoughts and get help on our forum (https://forum.image.sc/tags/biaflows), or write directly to our developer team at biaflows@neubias.org.

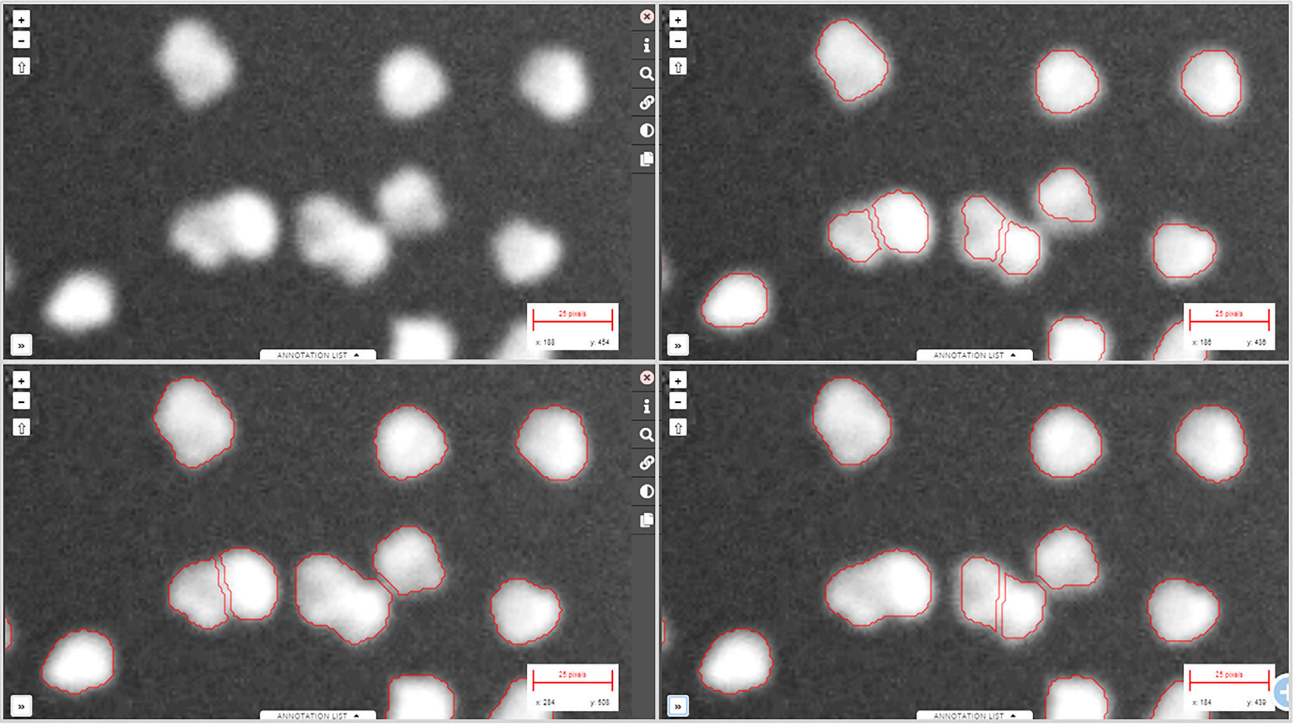

Figure 2. Synchronizing Image Viewers Displaying Different Workflow Results
Region from one of the sample images available in NUCLEI-SEGMENTATION problem (accessible from the BIAFLOWS online instance). Original image (upper left), same image overlaid with results from: custom ImageJ macro (upper right), custom CellProfiler pipeline (lower left), and custom Python script (lower right).

publications.[20] With this new design, benchmark metrics are automatically computed after every workflow run. BIAFLOWS can also be deployed on a local server to manage private images and workflows and to process images locally (Figure 3, BIAFLOWS local; Supplemental Experimental Procedures section 2). To simplify the coexistence of these different deployment scenarios, we developed migration tools (Supplementary Experimental Procedures section 5) to transfer content between existing BIAFLOWS instances (including the online instance described hereafter). Importantly, all content from any instance can be accessed programmatically through a RESTful interface, which ensures complete data accessibility and interoperability. Finally, for full flexibility, workflows can be downloaded manually from DockerHub to process local images independently of BIAFLOWS (Figure 3, standalone local; Supplemental Experimental Procedures section 5).

BIAFLOWS Online Curated Instance for Public Benchmarking

An online instance of BIAFLOWS is maintained by NEUBIAS and available at https://biaflows.neubias.org/ (Figure 3). This server is ready to host community contributions and is already populated with a substantial collection of annotated image datasets illustrating common BIA problems and several associated workflows to process these images (Table S1). Concretely, we integrated BIA workflows spanning nine important BIA problem classes illustrated by 15 image datasets imported from existing challenges (DIADEM,[21] Cell Tracking Challenge,[22] Particle Tracking Challenge,[23] Kaggle Data Science Bowl 2018[24]), created from synthetic data generators[25] (CytoPacq,[26] TREES toolbox,[27] Vascusynth,[28] SIMCEP[29]), or contributed by NEUBIAS members.[30] The following problem classes are currently represented: object detection/counting, object segmentation, and pixel classification (Figure 4); particle tracking, object tracking, filament network tracing, filament tree tracing, and landmark detection (Figure 5). To demonstrate the versatility of the platform we integrated 34 workflows, each targeting a specific software or programming language: ImageJ/FIJI macros and scripts,[31] Icy protocols,[32] CellProfiler pipelines,[33] Vaa3D plugins,[34] ilastik pipelines,[35] Octave scripts,[36] Jupyter notebooks,[15] and Python scripts leveraging Scikit-learn[37] for supervised learning algorithms, and Keras[38] or PyTorch[39] for deep learning. This list, although already extensive, is not limited, as BIAFLOWS core architecture enables one to seamlessly add other software as long as they fulfill minimal requirements (Supplemental Experimental Procedures section 3). To demonstrate the potential of the platform to perform open benchmarking, a case study has been performed with (and is available from) BIAFLOWS to compare workflows identifying nuclei in microscopy images. The content from the BIAFLOWS online instance (https://biaflows.neubias.org) can be viewed in read-only mode from the guest account, while the workflows can be launched from the sandbox server (https://biaflows-sandbox.neubias.org/). An extensive user guide and video tutorial are available online from the same URLs. To enhance their visibility, all workflows hosted in the system are also

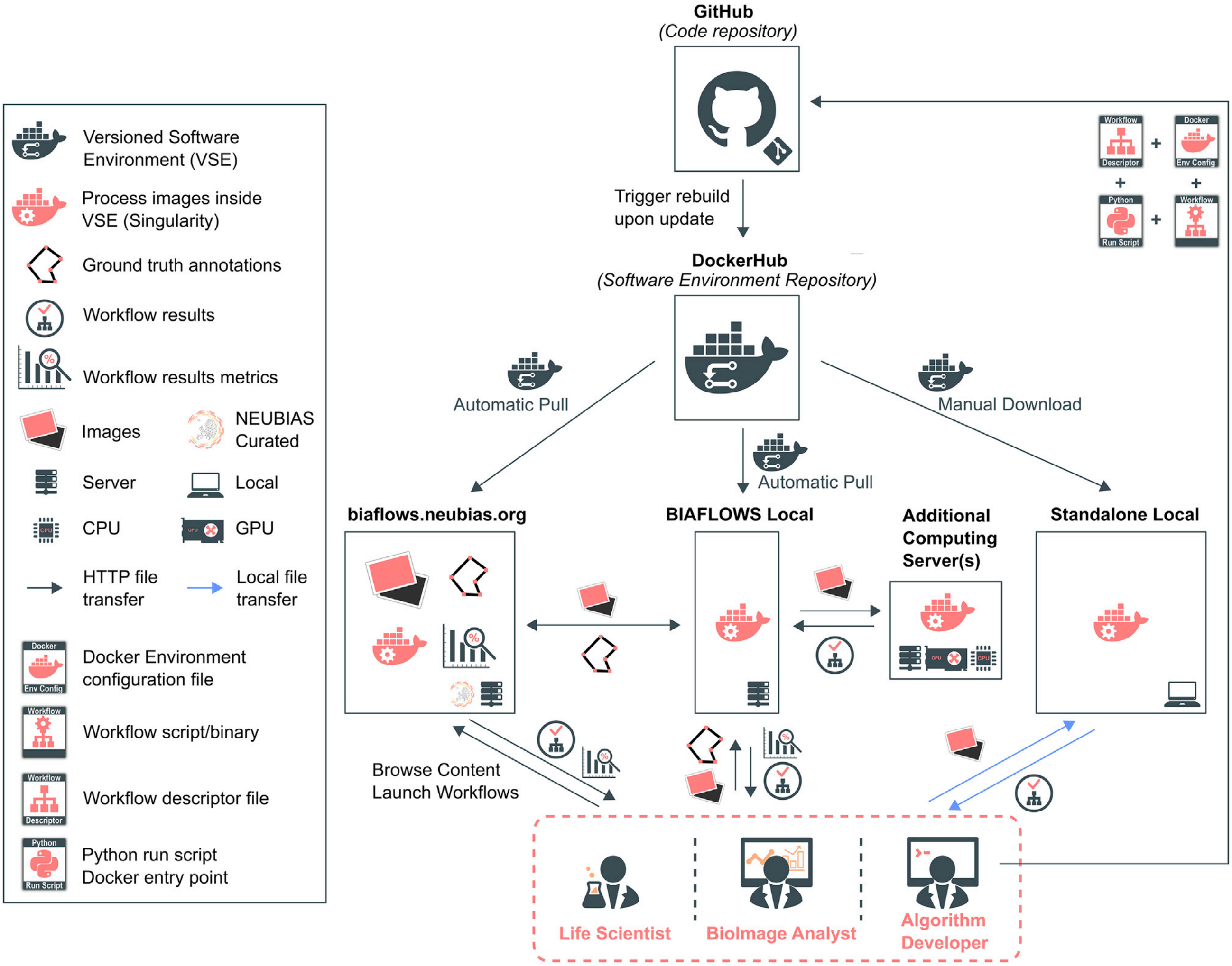

Figure 3. BIAFLOWS Architecture and Possible Deployment Scenarios

Workflows are hosted in a trusted source code repository (GitHub). Workflow (Docker) images encapsulate workflows together with their execution environments to ensure reproducibility. Workflow images are automatically built by a cloud service (DockerHub) whenever a new workflow is released or an existing workflow is updated from its trusted GitHub repository. Different BIAFLOWS instances monitor DockerHub and pull new or updated workflow images, which can also be downloaded to process local images without BIAFLOWS (Standalone Local).

referenced from NEUBIAS Bioimage Informatics Search Index (http://biii.eu/). BIAFLOWS online instance is fully extensible and, with minimal effort, interested developers can package their own workflows (Supplemental Experimental Procedures section 3) and make them available for benchmarking (Box 2). Similarly, following our guidelines (Supplemental Experimental Procedures section 2), scientists can make their images and ground-truth annotations available online through the online instance or through a local instance they manage (Box 2). Finally, all online content can be seamlessly migrated to a local BIAFLOWS instance (Supplemental Experimental Procedures section 5) for further development or to process local images.

To further increase the content currently available in BIAFLOWS online instance, calls for contribution will be shortly launched to gather more annotated microscopy images and encourage developers to package their own workflows. The support of new problem classes is also planned, for example, to benchmark the detection of blinking events in the context of super-resolution localization microscopy or the detection of landmark points for image registration. There is no limitation in using BIAFLOWS in other fields where image analysis is a critical step in extracting scientific results from images, for instance material or plant science and biomedical imaging.

Case Study: Comparing the Performance of Nuclei Segmentation by Classical Image Processing, Classical Machine Learning, and Deep-Learning Methods

To illustrate how to use BIAFLOWS for the open benchmarking of BIA workflows, we integrated seven nuclei segmentation workflows (Supplemental Experimental Procedures section 1). All content (images, ground-truth annotations, workflows, benchmark results) is readily accessible from the BIAFLOWS online instance. The workflows were benchmarked on two

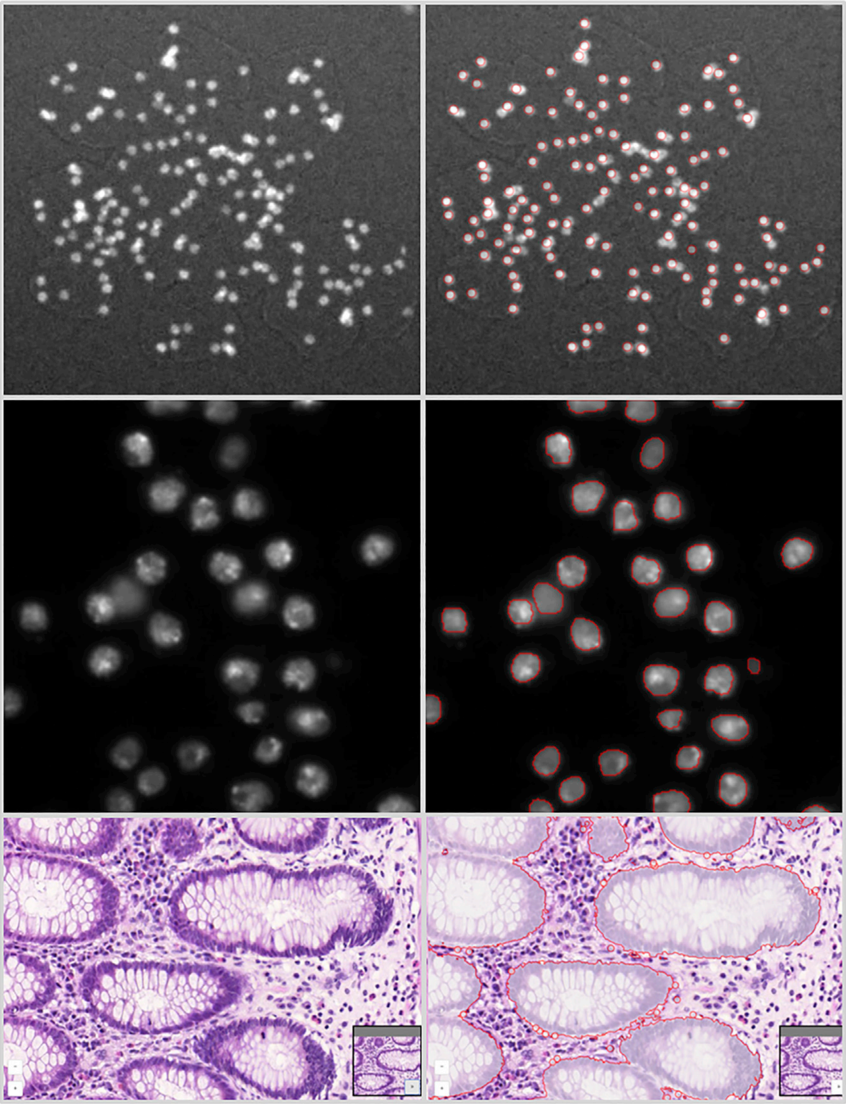

Figure 4. Sample Images from the BIAFLOWS Online Instance Illustrating Several BIA Problem Classes, and Results from Associated Workflows

Original image (left) and workflow results (right), from top to bottom: (1) spot detection in synthetic images (SIMCEP[29]); (2) nuclei segmentation in images from Kaggle Data Science Bowl 2018;[24] (3) pixel classification in images from 2015 MICCAI gland segmentation challenge.[40]

different image datasets: a synthetic dataset of ten images generated[29] for the purpose of this study, and a subset of 65 images from an existing nuclei segmentation challenge (Kaggle Data Science Bowl 2018[24]). The study was articulated in three parts: (1) evaluating the performance of three BIA workflows implementing classical methods to identify nuclei (synthetic dataset); (2) evaluating the performance of three ubiquitous deep-learning workflows on the same dataset; and (3) evaluating the performance of these deep-learning workflows (and a classical machine-learning workflow) on Kaggle Data Science Bowl 2018 (KDSB2018) subset. As a baseline, the classical workflows were manually tuned to obtain the best performance on the synthetic dataset while the machine-learning workflows were trained on generic nuclei image datasets with no further tuning for the synthetic dataset. Despite this, the deep-learning methods proved to be almost as accurate, or in some cases more accurate, than the best classical method (Tables S2 and S3). It was also evidenced that a set of benchmark metrics is generally to be favored over a single metric, since some widely used metrics only capture a single aspect of a complex problem. For instance, object segmentation does not only aim at accurately discriminating foreground from background pixels (assessed by DICE-like metrics) but overall at identifying independent objects (for instance to further measure their geometrical properties). Also, the visual inspection of workflow results proved useful in understanding the underlying errors evidenced by poor benchmark metrics results (Figure S1). All these features are readily available in BIAFLOWS, which swiftly enables to link workflow source code, benchmark metrics results, and visual results. The same methodology can be easily translated to other experiments.

DISCUSSION

BIAFLOWS addresses a number of critical requirements to foster open image analysis for life sciences: (1) sharing and visualizing annotated microscopy images illustrating commonly faced BIA problems; (2) sharing reproducible BIA workflows; (3) exposing workflow parameters and associated default values; (4) computing relevant benchmark metrics to compare workflows performance; and (5) providing a standard way to store, visualize, and share BIA workflows results. As such, BIAFLOWS is a central asset for biologists and bioimage analysts to leverage state-of-the-art bioimaging methods and efficiently reuse them in a different context. It is also a tool of choice for algorithm developers and challenge organizers to benchmark bioimage analysis workflows. Challenge participants traditionally reported workflow predictions on websites such as Kaggle and grand-challenge.org. The latter is currently developing a Docker-based mechanism (https://grand-challengeorg.readthedocs.io/en/latest/evaluation.html#) to package workflows (mostly coming from medical imaging), but these platforms do not offer a complete integrated web environment to host image datasets, automatically import workflows from open-source repositories, automate benchmark metric computation, and remotely visualize all results in a streamlined web interface such as BIAFLOWS. We believe BIAFLOWS could

CellPress
OPEN ACCESS

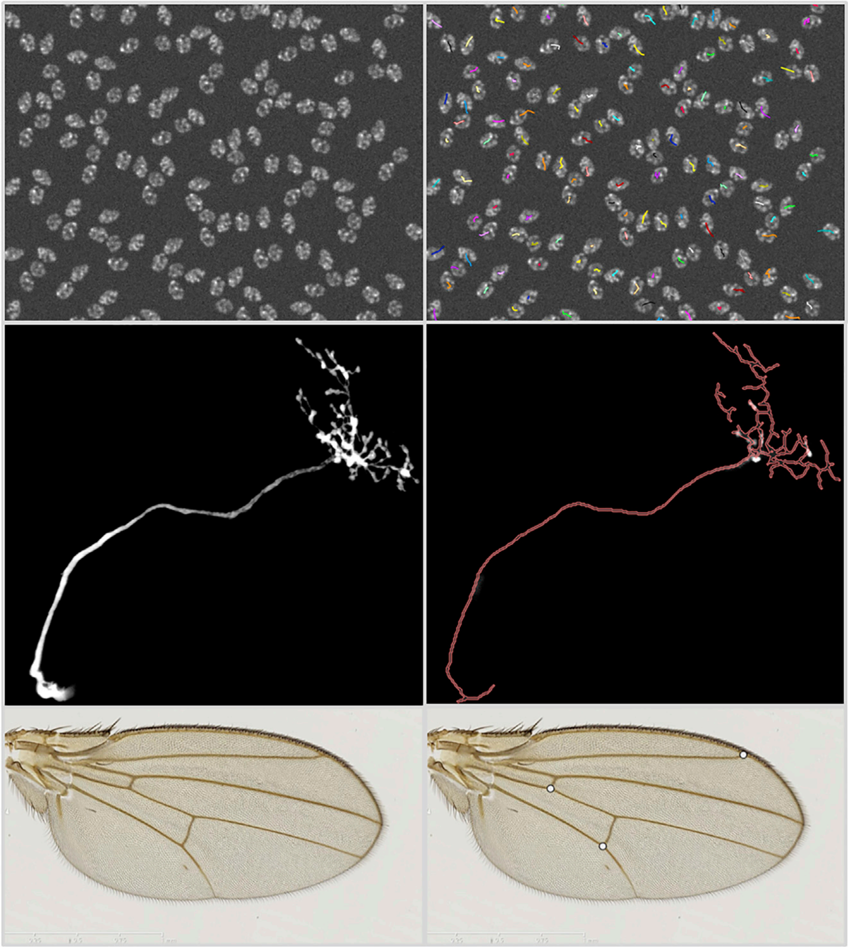

Figure 5. Sample Images from the BIAFLOWS Online Instance Illustrating Several BIA Problem Classes, and Results from Associated Workflows

Original image (left) and workflow results (right), from top to bottom: (1) particle tracking in synthetic time-lapse displaying non-dividing nuclei (CytoPACQ[26]), single frame + dragon-tail tracks; (2) neuron tree tracing in 3D image stacks from DIADEM challenge,[21] average intensity projection (left), traced skeleton z projection (dilated, red); (3) landmark detection in *Drosophila* wing images.[30]

be made interoperable with the grand-challenge.org Docker-based mechanism to package workflows, and used by challenge organizers as a fully integrated platform to automate benchmarking and share challenge results in a more reproducible way. Finally, BIAFLOWS provides a solution to authors willing to share online supporting data, methods, and results associated with their published scientific results.

With respect to sustainability and scalability, BIAFLOWS is backed by a team of senior bioimage analysts and software developers. The software is compatible with high-performance computing environments and is based on Cytomine architecture,[16] which has already proved itself capable of serving large datasets to many users simultaneously.[41] We invested a large amount of effort in documenting BIAFLOWS, and the online instance is ready to receive hundreds of new image datasets and workflows as community contributions (Box 2). To increase the content of BIAFLOWS online instance, we will briefly launch calls for contributions targeting existing

Box 2. How to Contribute to BIAFLOWS

- Scientists can contribute published annotated microscopy images to BIAFLOWS online instance. See ‘‘Problem classes, ground truth annotations and reported metrics’’ from the documentation portal for information on the expected images and ground-truth annotations formats, and contact us through the dedicated thread on https://forum.image.sc/tags/biaflows.
- To showcase a workflow in the BIAFLOWS online instance, developers can encapsulate their source code, test it on a local BIAFLOWS instance or BIAFLOWS sandbox server (https://biaflows-sandbox.neubias.org/), and open an issue in this GitHub repository: https://github.com/Neubias-WG5/SubmitToBiaflows. Follow ‘‘Creating a BIA workflow and adding it to a BIAFLOWS instance’’ from the documentation portal.
- Feature requests or bug reports can be posted to BIAFLOWS GitHub (https://github.com/neubias-wg5).
- Users can contribute to the documentation by submitting a pull request to https://github.com/Neubias-WG5/neubias-wg5.github.io.
- Any user can share data and results, e.g., accompanying scientific publications, via ‘‘Access BIAFLOWS from a Jupyter notebook’’ from the documentation portal or by directly linking the content of a BIAFLOWS instance.

BIAFLOWS problem classes. We propose that BIAFLOWS becomes a hub for BIA methods developers, bioimage analysts, and life scientists to share annotated datasets, reproducible BIA workflows, and associated results from benchmark and research studies. In future work, we will work toward interoperability with existing European image storage and workflow management infrastructures such as BioImage Archive,[42] https://www.eosc-life.eu/, and Galaxy,[15] and further improve the scalability and sustainability of the platform.

EXPERIMENTAL PROCEDURES

Resource Availability

Lead Contact

Further information and requests for resources should be directed to the Lead Contact, Sébastien Tosi (sebastien.tosi@irbbarcelona.org).

Materials Availability

No materials were used in this study.

Data and Code Availability

BIAFLOWS is an open-source project and its source code can be freely downloaded at https://github.com/Neubias-WG5.

All images and annotations described and used in this article can be downloaded from the BIAFLOWS online instance at https://biaflows.neubias.org/.

A sandbox server from which all workflows available in BIAFLOWS online instance can be launched remotely, and new workflows/datasets appended for testing are available at https://biaflows-sandbox.neubias.org/.

The documentation to install, use, and extend the software is available at https://neubias-wg5.github.io/.

SUPPLEMENTAL INFORMATION

Supplemental Information can be found online at https://doi.org/10.1016/j.patter.2020.100040.

ACKNOWLEDGMENTS

This project is funded by COST CA15124 (NEUBIAS). BIAFLOWS is developed by NEUBIAS (http://neubias.org) Workgroup 5, and it would not have been possible without the great support from the NEUBIAS vibrant community of bioimage analysts and the dedication of Julien Colombelli and Kota Miura in organizing this network. Local organizers of the Taggathons who have fostered the development of BIAFLOWS are also greatly acknowledged: Chong Zhang, Gabriel G. Martins, Julia Fernandez-Rodriguez, Peter Horvath, Bertrand Vernay, Aymeric Fouquier d'Hérouël, Andreas Girod, Paula Sampaio, Florian Levet, and Fabrice Cordelières. We also thank the software developers who helped us integrating external code, among others Jean-Yves Tinevez (Image Analysis Hub of Pasteur Institute) and Anatole Chessel (École Polytechnique). We thank the Cytomine SCRL FS for developing additional open-source modules and Martin Jones (Francis Crick Institute) and Pierre Geurts (University of Liège) for proofreading the manuscript and for their useful comments. R.V. was supported by ADRIC Pôle Mecatech Wallonia grant and U.R. by ADRIC Pôle Mecatech and DeepSport Wallonia grants. R. Marée was supported by an IDEES grant with the help of the Wallonia and the European Regional Development Fund. L.P. was supported by the Academy of Finland (grant 310552). M.M. was supported by the Czech Ministry of Education, Youth and Sports (project LTC17016). S.G.S. acknowledges the financial support of UEFISCDI grant PN-III-P1-1.1-TE-2016-2147 (CORIMAG). V.B. and P.P.-G. acknowledge the France-BioImaging infrastructure supported by the French National Research Agency (ANR-10-INBS-04).

AUTHOR CONTRIBUTIONS

S.T. and R. Marée conceptualized BIAFLOWS, supervised its implementation, contributed to all technical tasks, and wrote the manuscript. U.R. worked on the core implementation and web user interface of BIAFLOWS with contributions from G.M. and R.H. R. Mormont implemented several modules to interface bioimage analysis workflows and the content of the system. S.T., M.M., and D.Ü. implemented the module to compute benchmark metrics. S.T., V.B., R. Mormont, L.P., B.P., M.M., R.V., and L.A.S. integrated their own workflows or adapted existing workflows, and tested the system. S.T., D.Ü., O.G., and G.B. organized and collected content (image datasets, simulation tools). S.G.S., N.S., and P.P.-G. provided extensive feedback on BIAFLOWS and contributed to the manuscript. All authors took part in reviewing the manuscript.

DECLARATION OF INTERESTS

R Marée and R.H. are co-founders and members of the board of directors of the non-profit cooperative company Cytomine SCRL FS.

Received: February 14, 2020
Revised: April 4, 2020
Accepted: April 27, 2020
Published: June 3, 2020

REFERENCES

1. Ouyang, W., and Zimmer, C. (2017). The imaging tsunami: computational opportunities and challenges. Curr. Opin. Syst. Biol. *4*, 105–113.
2. Eliceiri, K.W., Berthold, M.R., Goldberg, I.G., Ibáñez, L., Manjunath, B.S., Martone, M.E., Murphy, R.F., Peng, H., Plant, A.L., Roysam, B., et al. (2012). Biological imaging software tools. Nat. Methods *9*, 697–710.
3. Carpenter, A.E., Kamentsky, L., and Eliceiri, K.W. (2012). A call for bioimaging software usability. Nat. Methods *9*, 666–670.
4. Schneider, C.A., Rasband, W.S., and Eliceiri, K.W. (2012). NIH Image to ImageJ: 25 years of image analysis. Nat. Methods *9*, 671–675.
5. Munafò, M.R., Nosek, B.A., Bishop, D.V.M., Button, K.S., Chambers, C.D., Percie du Sert, N., Simonsohn, U., Wagenmakers, E.-J., Ware, J.J., and Ioannidis, J.P.A. (2017). A manifesto for reproducible science. Nat. Hum. Behav. *1*, 0021.
6. Hutson, M. (2018). Artificial intelligence faces reproducibility crisis. Science *359*, 725–726.
7. Ellenberg, J., Swedlow, J.R., Barlow, M., Cook, C.E., Sarkans, U., Patwardhan, A., Brazma, A., and Birney, E. (2018). A call for public archives for biological image data. Nat. Methods *15*, 849–854.
8. Allan, C., Burel, J.M., Moore, J., Blackburn, C., Linkert, M., Loynton, S., Macdonald, D., Moore, W.J., Neves, C., Patterson, A., et al. (2012). OMERO: flexible, model-driven data management for experimental biology. Nat. Methods *9*, 245–253.
9. Kvilekval, K., Fedorov, D., Obara, B., Singh, A., and Manjunath, B.S. (2010). Bisque: a platform for bioimage analysis and management. Bioinformatics *26*, 544–552.
10. Williams, E., Moore, J., Li, S.W., Rustici, G., Tarkowska, A., Chessel, A., Leo, S., Antal, B., Ferguson, R.K., Sarkans, U., et al. (2017). Image Data Resource: a bioimage data integration and publication platform. Nat. Methods *14*, 775–781.
11. Vandewalle, P. (2012). Code sharing is associated with research impact in image processing. Comput. Sci. Eng. *14*, 42–47.
12. Maier-Hein, L., Eisenmann, M., Reinke, A., Onogur, S., Stankovic, M., Scholz, P., Arbel, T., Bogunovic, H., Bradley, A.P., Carass, A., et al. (2018). Why rankings of biomedical image analysis competitions should be interpreted with care. Nat. Commun. *9*, 5217.
13. Meijering, E., Carpenter, A., Peng, H., Hamprecht, F.A., and Olivo-Marin, J.C. (2016). Imagining the future of bioimage analysis. Nat. Biotechnol. *34*, 1250–1255.
14. Perkel, J.M. (2018). A toolkit for data transparency takes shape. Nature *560*, 513–515.
15. Grüning, B.A., Rasche, E., Rebolledo-Jaramillo, B., Eberhard, C., Houwaart, T., Chilton, J., Coraor, N., Backofen, R., Taylor, J., and Nekrutenkoet, A. (2017). Jupyter and Galaxy: easing entry barriers into

complex data analyses for biomedical researchers. PLoS Comput. Biol. *13*, e1005425.

16. Marée, R., Rollus, L., Stévens, B., Hoyoux, R., Louppe, G., Vandaele, R., Begon, J.M., Kainz, P., Geurts, P., and Wehenkel, L. (2016). Collaborative analysis of multi-gigapixel imaging data with Cytomine. Bioinformatics *32*, 1395–1401.
17. Glatard, T., Kiar, G., Aumentado-Armstrong, T., Beck, N., Bellec, P., Bernard, R., Bonnet, A., Brown, S.T., Camarasu-Pop, S., Cervenansky, F., et al. (2018). Boutiques: a flexible framework to integrate command-line applications in computing platforms. GigaScience *7*, giy016.
18. Kurtzer, G.M., Sochat, V., and Bauer, M.W. (2017). Singularity: scientific containers for mobility of compute. PLoS One *12*, e0177459.
19. Yoo, A., Jette, M., and Grondona, M. (2003). SLURM: simple Linux utility for resource management, job scheduling strategies for parallel processing. Lect. Notes Comput. Sci. *2862*, 44–60.
20. Kozubek, M. (2016). Challenges and benchmarks in bioimage analysis. Adv. Anat. Embryol. Cell Biol. *219*, 231–262.
21. Brown, K.M., Barrionuevo, G., Canty, A.J., De Paola, V., Hirsch, J.A., Jefferis, G.S., Lu, J., Snippe, M., Sugihara, I., and Ascoli, G.A. (2011). The DIADEM data sets: representative light microscopy images of neuronal morphology to advance automation of digital reconstructions. Neuroinformatics *9*, 143–157.
22. Ulman, V., Maška, M., Magnusson, K.E.G., Ronneberger, O., Haubold, C., Harder, N., Matula, P., Matula, P., Svoboda, D., Radojevic, M., et al. (2017). An objective comparison of cell-tracking algorithms. Nat. Methods *14*, 1141–1152.
23. Chenouard, N., Smal, I., de Chaumont, F., Maška, M., Sbalzarini, I.F., Gong, Y., Cardinale, J., Carthel, C., Coraluppi, S., Winter, M., et al. (2014). Objective comparison of particle tracking methods. Nat. Methods *11*, 281–289.
24. Caicedo, J.C., Goodman, A., Karhohs, K.W., Cimini, B.A., Ackerman, J., Haghighi, M., Heng, C., Becker, T., Doan, M., McQuin, C., et al. (2019). Nucleus segmentation across imaging experiments: the 2018 data science bowl. Nat. Methods *16*, 1247–1253.
25. Svoboda, D., Kozubek, M., and Stejskal, S. (2009). Generation of digital phantoms of cell nuclei and simulation of image formation in 3D image cytometry. Cytometry A *75*, 494–509.
26. Wiesner, D., Svoboda, D., Maška, M., and Kozubek, M. (2019). CytoPacq: a web-interface for simulating multi-dimensional cell imaging. Bioinformatics *35*, 4531–4533.
27. Cuntz, H., Forstner, F., Borst, A., and Häusser, M. (2010). One rule to grow them all: a general theory of neuronal branching and its practical application. PLoS Comput. Biol. *6*, e1000877.
28. Jassi, P., and Hamarneh, G. (2011). VascuSynth: vascular tree synthesis software. Insight J http://hdl.handle.net/10380/3260.
29. Lehmussola, A., Ruusuvuori, P., Selinummi, J., Huttunen, H., and Yli-Harja, O. (2007). Computational framework for simulating fluorescence microscope images with cell populations. IEEE Trans. Med. Imaging *26*, 1010–1016.
30. Vandaele, R., Aceto, J., Muller, M., Péronnet, F., Debat, V., Wang, C.W., Huang, C.T., Jodogne, S., Martinive, P., Geurts, P., et al. (2018). Landmark detection in 2D bioimages for geometric morphometrics: a multi-resolution tree-based approach. Sci. Rep. *8*, 538.
31. Schneider, C.A., Rasband, W.S., and Eliceiri, K.W. (2012). NIH Image to ImageJ: 25 years of image analysis. Nat. Methods *9*, 671–675.
32. de Chaumont, F., Dallongeville, S., Chenouard, N., Hervé, N., Pop, S., Provoost, T., Meas-Yedid, V., Pankajakshan, P., Lecomte, T., Le Montagner, Y., et al. (2012). Icy: an open bioimage informatics platform for extended reproducible research. Nat. Methods *9*, 690–696.
33. McQuin, C., Goodman, A., Chernyshev, V., Kamentsky, L., Cimini, B.A., Karhohs, K.W., Doan, M., Ding, L., Rafelski, S.M., Thirstrup, D., et al. (2018). CellProfiler 3.0: next-generation image processing for biology. PLoS Biol. *16*, e2005970.
34. Peng, H., Ruan, Z., Long, F., Simpson, J.H., and Myers, E.W. (2010). V3D enables real-time 3D visualization and quantitative analysis of large-scale biological image data sets. Nat. Biotechnol. *28*, 348–353.
35. Berg, S., Kutra, D., Kroeger, T., Straehle, C.N., Kausler, B.X., Haubold, C., Schiegg, M., Ales, J., Beier, T., Rudy, M., et al. (2019). ilastik: interactive machine learning for (bio)image analysis. Nat. Methods *16*, 1226–1232.
36. Eaton, J.W., Bateman, D., Hauberg, S., and Wehbring, R. (2016). GNU Octave Version 4.2.0 Manual: A High-Level Interactive Language for Numerical Computations (Free Software Foundation). https://octave.org/doc/octave-4.2.0.pdf.
37. Pedregosa, F., Varoquaux, G., Gramfort, A., Michel, V., Thirion, B., Grisel, O., Blondel, M., Prettenhofer, P., Weiss, R., Dubourg, V., et al. (2011). Scikit-learn: machine learning in Python. J. Mach. Learn. Res. *12*, 2825–2830.
38. Chollet, F. (2017). Deep Learning with Python (Manning).
39. Paszke, A., Gross, S., Chintala, S., Chanan, G., Yang, E., DeVito, Z., Lin, Z., Desmaison, A., Antiga, L., Lerer, A. (2017). Automatic Differentiation in PyTorch. NIPS Autodiff Workshop, 2017.
40. Sirinukunwattana, K., Pluim, J.P.W., Chen, H., Qi, X., Heng, P.A., Guo, Y.B., Wang, L.Y., Matuszewski, B.J., Bruni, E., Sanchez, U., et al. (2017). Gland segmentation in colon histology images: the glas challenge contest. Med. Image Anal. *35*, 489–502.
41. Multon, S., Pesesse, L., Weatherspoon, A., Florquin, S., Van de Poel, J.F., Martin, P., Vincke, G., Hoyoux, R., Marée, R., Verpoorten, D., et al. (2018). A Massive Open Online Course (MOOC) on practical histology: a goal, a tool, a large public! Return on a first experience. Ann. Pathol. *38*, 76–84.
42. Ellenberg, J., Swedlow, J.R., Barlow, M., Cook, C.E., Sarkans, U., Patwardhan, A., Brazma, A., and Birney, E. (2018). A call for public archives for biological image data. Nat. Methods *15*, 849–854.

PATTER, Volume *1*

Supplemental Information

BIAFLOWS: A Collaborative Framework to Reproducibly Deploy and Benchmark Bioimage Analysis Workflows

Ulysse Rubens, Romain Mormont, Lassi Paavolainen, Volker Bäcker, Benjamin Pavie, Leandro A. Scholz, Gino Michiels, Martin Maška, Devrim Ünay, Graeme Ball, Renaud Hoyoux, Rémy Vandaele, Ofra Golani, Stefan G. Stanciu, Natasa Sladoje, Perrine Paul-Gilloteaux, Raphaël Marée, and Sébastien Tosi

Problem class	Problem	Workflow repository	BISE link
1. Object detection / 2. Object counting	SPOT-COUNTING-2D	W_SpotDetection-IJ	http://biii.eu/spot-detection-imagej
	SPOT-DETECTION-2D	W_SpotDetection-Icy	http://biii.eu/spot-detection-icy
		W_SpotDetection-Dmap-IJ	http://biii.eu/node/1603
	SPOT-COUNTING-3D	W_SpotDetection3D-IJ	http://biii.eu/node/1458
	SPOT-DETECTION-3D	W_SpotDetection3D-Icy	http://biii.eu/node/1604
		W_SpotDetection3D-Hessian-IJ	http://biii.eu/spot-detection-3d-hessian-imagej
3. Object segmentation	NUCLEI-SEGMENTATION	W_NucleiSegmentation-ImageJ	http://biii.eu/nuclei-segmentation-2d-imagej
		W_NucleiSegmentation-CellProfiler	https://biii.eu/nuclei-segmentation-cellprofiler
		W_NucleiSegmentation-Python	http://biii.eu/nuclei-segmentation-python
		W_NucleiSegmentation-MaskRCNN	https://biii.eu/node/1487
		W_NucleiSegmentation-DeepCell	http://biii.eu/node/1607
		W_NucleiSegmentation-UNet	http://biii.eu/node/1608
	DATA-SCIENCE-BOWL-2018	W_NucleiSegmentation-MaskRCNN	https://biii.eu/node/1487
		W_NucleiSegmentation-UNet	http://biii.eu/node/1608
		W_NucleiSegmentation-ilastik	https://biii.eu/nuclei-segmentation-ilastik
	NUCLEI-SEGMENTATION-3D	W_NucleiSegmentation3D-ImageJ	http://biii.eu/nuclei-segmentation-3d-imagej
		W_NucSeg3DThr-ImageJ	http://biii.eu/node/1609
		W_NucleiSegmentation3D-ilastik	http://biii.eu/node/1610
4. Pixel classification	GLAND-SEGMENTATION	W_PixCla-UNet-GlaS	https://biii.eu/pixel-classification-glas-challenge-unet
5. Particle tracking	NUCLEI-TRACKING-NODIVISION	W_NucleiTracking-ImageJ	https://biii.eu/nuclei-tracking-imagej
		W_LogPartTrack_IJ	http://biii.eu/node/1611
		W_NucleiTrackingTrackmate-IJ	http://biii.eu/nuclei-tracking-trackmate
6. Object tracking	NUCLEI-TRACKING-DIVISION	W_ObjectTracking-ImageJ	http://biii.eu/node/1614
		W_ObjectTracking-Octave	http://biii.eu/node/1615
		W_ObjectTraking-MU-Lux-CZ	http://biii.eu/node/1616
	NUCLEI-TRACKING-3D	NO WORKFLOW YET	
7. Filament tree network tracing	NEURON-TRACING-3D	W_NeuronTracing_vaa3d	https://biii.eu/app-all-path-pruning
		W_NeuronTracing_vaa3d_app2	http://biii.eu/node/1617
		W_NeuronTracing3D_Rivuletpy	http://biii.eu/node/1618
		W_NeuronTracing_vaa3d_most	http://biii.eu/node/1620
		W_NeuronTracing_vaa3d_fastmarchi	http://biii.eu/node/1623
	NEURON-TRACING-TREES-3D	W_NeuronTracing_vaa3d	https://biii.eu/app-all-path-pruning
		W_NeuronTracing_vaa3d_app2	http://biii.eu/node/1617
		W_NeuronTracing_vaa3d_most	http://biii.eu/node/1620
		W_NeuronTracing_vaa3d_fastmarchi	http://biii.eu/node/1623
8. Filament network tracing	VESSEL-TRACING-3D	W_FilamentTracing3D-ImageJ	http://biii.eu/node/1453
		W_FilamentTracing3D-Tub-IJ	http://biii.eu/node/1621
		W_FilamentTracing3D-LocThr-IJ	http://biii.eu/node/1622
9. Landmark detection	LANDMARKS-DROSO	W_LandmarkDetect-ML-MSET-Pred	https://biii.eu/landmark-detection-mset-models-prediction
		W_LandmarkDetect-ML-LC-Pred	https://biii.eu/landmark-detection-lc-models-prediction
		W_LandmarkDetect-ML-DMBL-Pred	https://biii.eu/node/1485

Table S1. BIA problems and workflows currently available from BIAFLOWS online instance. Related to Figure 1. Problem class: type of image analysis problem. Problem: concrete BIA problem, as listed from BIAFLOWS > Problems webpage. Workflows repository: code repository, as linked from BIAFLOWS > Workflows webpage (and stored at https://github.com/Neubias-WG5). BISE link: workflow webpage on BISE, NEUBIAS Bioimage Informatics Search Engine.

Name	mAP	FO	DICE	AHD
w_nucleisegmentation_maskrcnn	**0.606**	0.797	0.915	0.224
w_nucleisegmentation_unet	0.596	0.795	**0.922**	0.361
w_nucleisegmentation-imagej	0.593	**0.821**	0.912	**0.119**
w_nucleisegmentation_deepcell	0.58	0.768	0.917	0.416
w_nucleisegmentation-python	0.549	0.763	0.904	0.322
w_nucleisegmentation_cellprofiler	0.482	0.724	0.872	0.148

Table S2. Benchmark metrics results for the six nuclei segmentation workflows (SIMCEP dataset). Best results in bold, green highlights results significantly better than average, and red highlights results significantly worse than average.

Name	mAP	FO	DICE	AHD
w_nucleisegmentation_maskrcnn	**0.394**	**0.625**	**0.798**	**2.702**
w_nucleisegmentation_unet	0.282	0.542	0.754	8.485
w_nucleisegmentation_ilastik	0.208	0.502	0.725	3.843

Table S3. Benchmark metrics results for the three nuclei segmentation machine learning workflows (DSB dataset). Best results in bold, green highlights results significantly better than average, and red highlights results significantly worse than average.

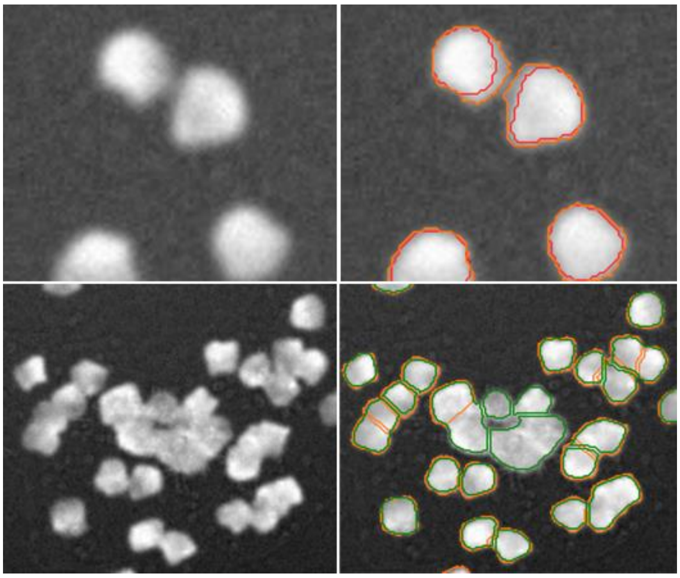

Figure S1. Some workflow results (SIMCEP dataset). Cropped out regions from original images (left), same regions with workflow results overlay (right), top: U-NET (red) and CellProfiler (orange), bottom: DeepCell (green) and CellProfiler (orange).

Supplemental Experimental Procedures

Section 1. Case study: Nuclei segmentation

In this section, we present two simple comparison experiments showcasing BIAFLOWS features. Both experiments can be fully reproduced from BIAFLOWS online instance.

Datasets (available from BIAFLOWS > Problems)

1. **BIAFLOWS NUCLEI-SEGMENTATION (SIMCEP),** Related to Figure 2
   10 synthetic grayscale images simulating widefield fluorescence microscopy images created by[1]. The images exhibit strong non-uniform illumination, saturation, and some nuclei are heavily clustered.

2. **BIAFLOWS DATA-SCIENCE-BOWL-2018 (DSB)**, Related to Figure 4
   65 RGB images from Data Science Bowl 2018 challenge dataset[2], exhibiting heterogeneous stained nuclei samples imaged from various microscopy modalities.

Workflows (available from BIAFLOWS > Workflows), Related to Figure 2 and Figure S1

**Name**	**Pre-processing**	**Classification**	**Mask post-processing**
w_nucleisegmentation-imagej	Laplacian of Gaussian	Global threshold (user defined)	Binary watershed from distance map
w_nucleisegmentation-python	Gaussian blur	Adaptive threshold (local mean)	Binary watershed from smoothed distance map, remove small objects
w_nucleisegmentation_cellprofiler	Illumination correction	Global threshold (3 class Otsu's method)	Hole filling, Binary watershed from smoothed distance map, remove small and large objects
w_nucleisegmentation_ilastik	None	2 class pixel random forest classifier[1] (trained on 15 images from DSB2018 training set 1)	Hole filling, binary watershed from smoothed distance map, remove small objects
w_nucleisegmentation_maskrcnn[3]	None	Pre-trained Mask R-CNN model from[4] (trained on 670 images from DSB2018 training set 1)	None, but classifier accounts for object geometry
w_nucleisegmentation_unet[5]	None	3 class pixel classifier U-NET model trained[2] on 670 images from DSB2018 training set 1	Hole filling, binary watershed from smoothed distance map, remove small and edge touching objects
w_nucleisegmentation_deepcell	None	Pre-trained DeepCell 1.0 model from[6] (trained on mammalian nuclei images)	Hole filling, binary watershed from smoothed distance map, remove small and edge touching objects

[1] Features: Gaussian Smoothing, Laplacian of Gaussian, Gaussian Gradient Magnitude, Difference of Gaussians, Structure Tensor Eigenvalues, Hessian of Gaussian Eigenvalues. Sigma: 0.7, 1.0, 1.6 and 3.5.

[2] lr = 1e-4, 15 epochs (500 steps), batch size: 10, loss: weighted cross-entropy, optimizer: RMSprop.

Benchmark metrics (reported in BIAFLOWS > Problems > Workflow runs), Related to Figure 1

Dice coefficient (DICE, 0-1): normalized overlap between ground truth and prediction binary masks. DICE is equal to 1 only for perfect segmentation.

Average Hausdorff Distance (AHD, >=0): average distance between object pixels in ground truth (/prediction) masks and closest object pixels in prediction (/ground truth) masks. AHD is equal to 0 only for perfect segmentation.

Fraction overlap (FO, 0-1): 0.5 fraction overlap can be interpreted as "on average the overlap of a predicted object with the ground truth object with largest overlap is half the area of the larger of these two objects". This would for instance happen if objects are either systematically split into two identical objects or merged by pair. FO is equal to 1 only for perfect segmentation.

Mean Average Precision (mAP, 0-1)[2]: IoU (Intersection over Union) between predicted and ground-truth objects are computed. IoU are then compared to 10 thresholds (0.5, 0.55 ... 0.95) and, if greater, the object is set as true positive (TP) for that threshold. Precision is computed as P = TP / (TP + FP + FN) for each threshold, where FP = number of predicted objects - TP and FN = number of ground-truth objects - TP. Precision is finally averaged out for all objects and images. mAP is equal to 1 only for perfect segmentation.

Benchmark metrics results (available from BIAFLOWS > Problems > Workflow runs)

Synthetic nuclei image dataset (SIMCEP)

The aim of this first experiment is two-fold: 1) comparing the performance of three nuclei segmentation workflows implementing classical image analysis methods, and which parameters were manually tuned for the SIMCEP dataset, 2) comparing the performance of these workflows to three Deep Learning (DL) workflows trained on generic nuclei microscopy image datasets. All content, including workflow source code, workflows parameters used for this experiment, benchmark metrics results, and workflow visual results, are available online: https://biaflows.neubias.org/#/project/5955/analysis. The benchmark metrics results of this experiment are summarized in Table S2.

For object segmentation, mean Average Precision (mAP, from Data Science Bowl 2018 challenge) is one of the most relevant metric (main metric) since it assesses the ability of a workflow to identify independent nuclei. Despite the fact that they were trained on generic nuclei datasets, DL workflows achieve among the best mAP. Clearly, DICE and AHD only reflects one aspect of the problem, concretely the ability of a workflow to classify pixels as being part of an object (DICE coefficient), or in its vicinity (Average Hausdorff Distance, AHD). As a consequence, DICE metric is poorly discriminative for this experiment as it does not account for erroneous object merging or splitting. Still, comparing the best (U-NET) and worse workflow (CellProfiler) for DICE metric, it is apparent that this latter tend to overestimate the extension of the nuclei by including part of the blur surrounding them. AHD is rather poorly informative for this experiment, the only two workflows achieving significantly higher AHD are the only ones excluding nuclei touching the edges (these objects contribute to significantly increasing AHD).

A simple workflow consisting of Laplacian of Gaussian (LoG) pre-filtering followed by user defined global thresholding (ImageJ workflow) and binary watershed achieves close or better than the DL workflows, and it is the most successful classical method represented. This is probably since 1) the size of the nuclei is rather uniform for this dataset (LoG radius can be fine-tuned), 2) the images suffer from heavy non-uniform illumination (LoG is insensitive to smooth intensity variations) and 3) some nuclei are heavily clustered (LoG displays a strong response around blobs helping to split them apart). Fine-tuned local adaptive thresholding (Python workflow) and illumination correction followed by automatic global thresholding (CellProfiler workflow), both followed by similar post-processing, achieve lower mAP. CellProfiler workflow especially does not manage to identify independent nuclei in heavily clustered regions. Finally, the results from the relatively simpler Fraction Overlap (FO) metric (implemented for the first time in BIAFLOWS) correlates quite well with the more complex mAP metric results.

Real microscopy nuclei dataset (DSB)

The aim of this experiment is two-fold: 1) comparing some of the previous deep learning workflows on a real microscopy test set similar to their training sets, 2) comparing their performance to a more classical machine learning workflow (Ilastik, using local feature extraction and random forest pixel classification). All content, including workflow source code, workflows parameters used for this experiment, benchmark metrics results, and workflow visual results, are available online: https://biaflows.neubias.org/_#/project/12182234/analysis. The benchmark metrics results of this experiment are summarized in Table S3.

Overall, all metric results are significantly worse than for the previous experiment, showing that this dataset is far more challenging. Due to the complexity and heterogeneity of this dataset, classical workflows were not evaluated. DeepCell 1.0 workflow was also excluded since it was trained on a dataset too different from DSB dataset (grayscale, fluorescence microscopy images). Mask R-CNN clearly outperforms other ML workflows (for all four metrics), suggesting that a strategy consisting in classifying pixels and splitting apart clusters of objects is not as successful as direct object detection and segmentation for complex images. It is also apparent that more classical pixel classification ML techniques (Ilastik) are not able to deal with class heterogeneity as well as DL methods. As a common practice, Ilastik developers actually recommend to enforce in class homogeneity, which cannot be checked for this dataset. Also, Ilastik was only trained from 15 representative images of the whole training set (65 images) since the size of the model quickly becomes unpractical for growing image numbers (the software is originally designed for sparse hand annotations).

Discussion

This simple case study showcased some important BIAFLOWS features and the importance of using a set of benchmark metrics (as opposed to a single metric). It also confirmed the versatility of Deep Learning methods, both to deal with heterogeneous datasets[2], and to generalize to datasets completely different than their training sets (even without transfer learning or other advanced strategies). The simple Fraction Overlap (FO) metric that we implemented for BIAFLOWS correlates quite well with the more complex mean Average Precision (mAP) proposed for the Data Science Bowl challenge. These two metrics are undoubtedly the best of the four metrics to capture the ability of the workflows to identify independent objects. mAP metric seems slightly more sensitive than the simpler FO metric, but this latter is easier to interpret in terms of the average normalized overlap between predicted and ground truth objects. DICE and AHD metrics, while bringing complementary information and orientation on how to interpret difference in performance, are not sufficient to assess true accuracy since they do not account for object splitting and merging errors.
Importantly, visual inspection was instrumental to conclude on the actual shortcomings of the workflows. BIAFLOWS enables to browse benchmark metrics results both as agglomerated statistics and per image, and it is also easy to jump from benchmark metrics results to the original images or compare visual workflow results side by side. Finally, all results are publically available (including workflows source code) and can be reproduced online, which contrasts with common benchmarking publication practices, e.g.[2], where the code provided sometimes requires expert setup to run locally.

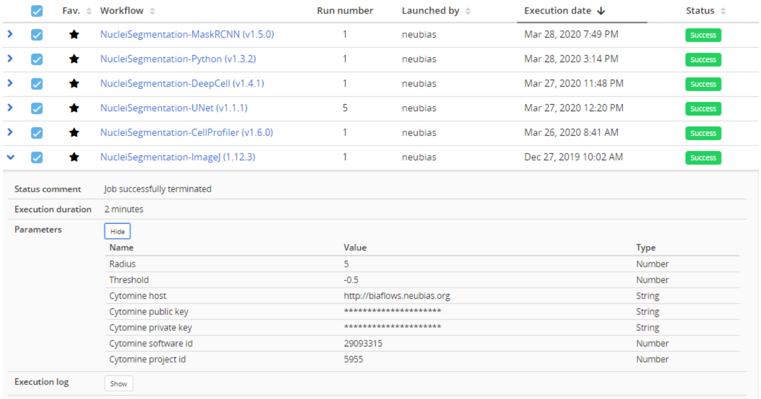

BIAFLOWS workflow runs for SIMCEP dataset. For every run, the parameters used and the execution log can be retrieved from drop down tabs.

Aggregated results | Detailed results per image

Workflow run	Fraction Overlap Pred		Dice coefficient		Average Hausdorff distance		Mean Average Precision	
	AVG	SD	AVG	SD	AVG	SD	AVG	SD
★ NucleiSegmentation-MaskRCNN (v1.5.0) #1 on Mar 28, 2020 7:49 PM	0.797	0.061	0.915	0.018	0.224	0.25	0.606	0.06
★ NucleiSegmentation-Python (v1.3.2) #1 on Mar 28, 2020 3:14 PM	0.763	0.041	0.904	0.008	0.322	0.164	0.549	0.064
★ NucleiSegmentation-DeepCell (v1.4.1) #1 on Mar 27, 2020 11:48 PM	0.768	0.075	0.917	0.007	0.416	0.208	0.58	0.065
★ NucleiSegmentation-UNet (v1.1.1) #5 on Mar 27, 2020 12:20 PM	0.795	0.05	0.922	0.012	0.361	0.191	0.596	0.07
★ NucleiSegmentation-CellProfiler (v1.6.0) #1 on Mar 26, 2020 8:41 AM	0.722	0.048	0.872	0.012	0.24	0.183	0.482	0.058
★ NucleiSegmentation-ImageJ (1.12.3) #1 on Dec 27, 2019 10:02 AM	0.821	0.049	0.912	0.012	0.119	0.044	0.593	0.07

Aggregated results | Detailed results per image

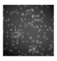

Img_0005_Nuc_Norm.tif

Workflow run	Fraction Overlap Pred	Dice coefficient	Average Hausdorff distance	Mean Average Precision
★ NucleiSegmentation-MaskRCNN (v1.5.0) #1 on Mar 28, 2020 7:49 PM	0.815	0.924	0.106	0.603
★ NucleiSegmentation-Python (v1.3.2) #1 on Mar 28, 2020 3:14 PM	0.772	0.901	0.556	0.558
★ NucleiSegmentation-DeepCell (v1.4.1) #1 on Mar 27, 2020 11:48 PM	0.774	0.911	0.731	0.575
★ NucleiSegmentation-UNet (v1.1.1) #5 on Mar 27, 2020 12:20 PM	0.796	0.915	0.648	0.592
★ NucleiSegmentation-CellProfiler (v1.6.0) #1 on Mar 26, 2020 8:41 AM	0.731	0.867	0.161	0.47
★ NucleiSegmentation-ImageJ (1.12.3) #1 on Dec 27, 2019 10:02 AM	0.839	0.913	0.109	0.605

BIAFLOWS workflow metric results table for the SIMCEP dataset. Top: aggregated (all images, metrics average and standard deviation), bottom: metrics per image.

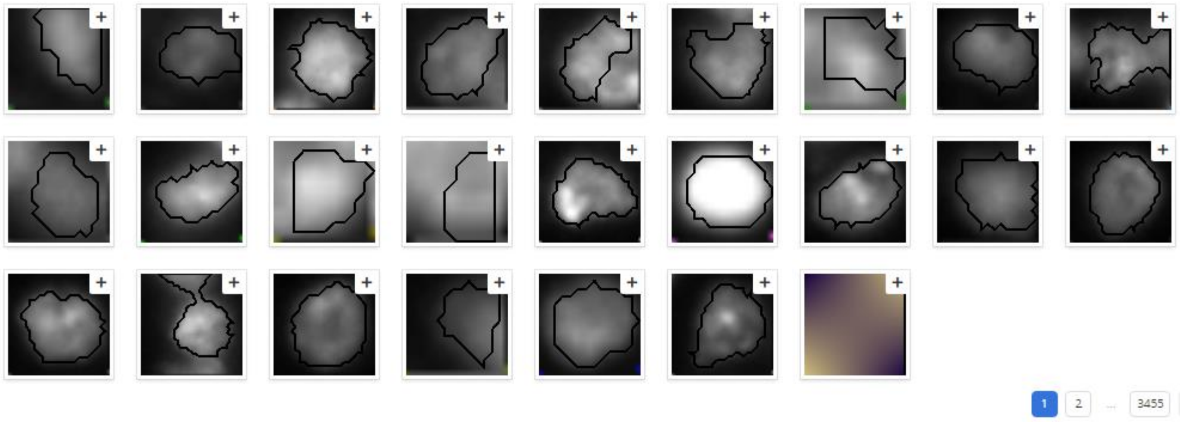

BIAFLOWS gallery showing cells segmented by U-NET (first 25) from DSB dataset. Each individual object can be clicked and visualized in context.

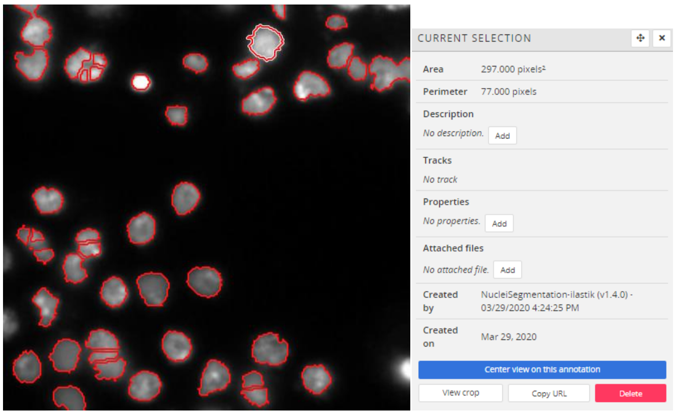

BIAFLOWS image viewer displaying one image from DSB dataset and cells segmented by U-Net. Some information on a selected cell (highlighted) is displayed, this cell is the third cell displayed in the gallery figure.

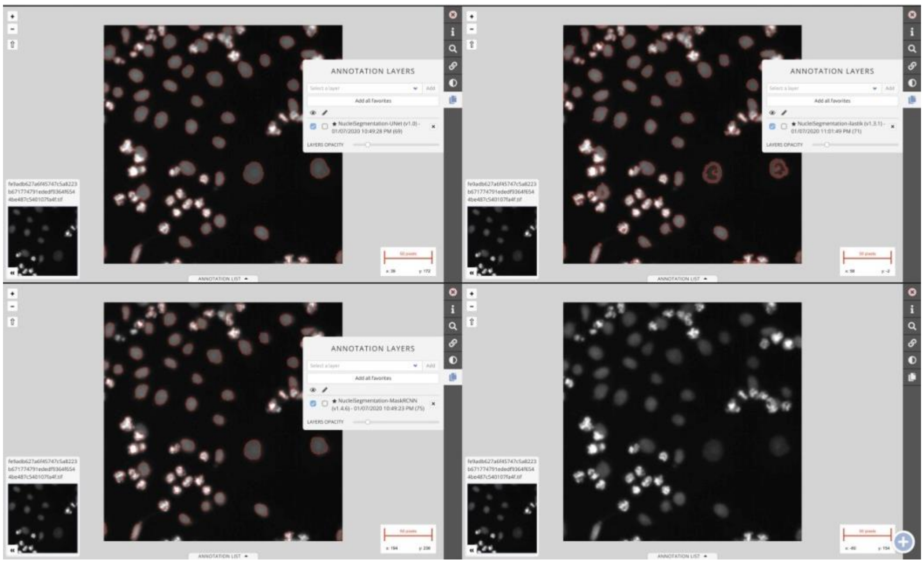

BIAFLOWS image viewer side-by-side comparison of segmented cells by three workflows of one DSB image. Top left: U-NET, top right: Ilastik, bottow left: Mask R-CNN, bottom right: original image.

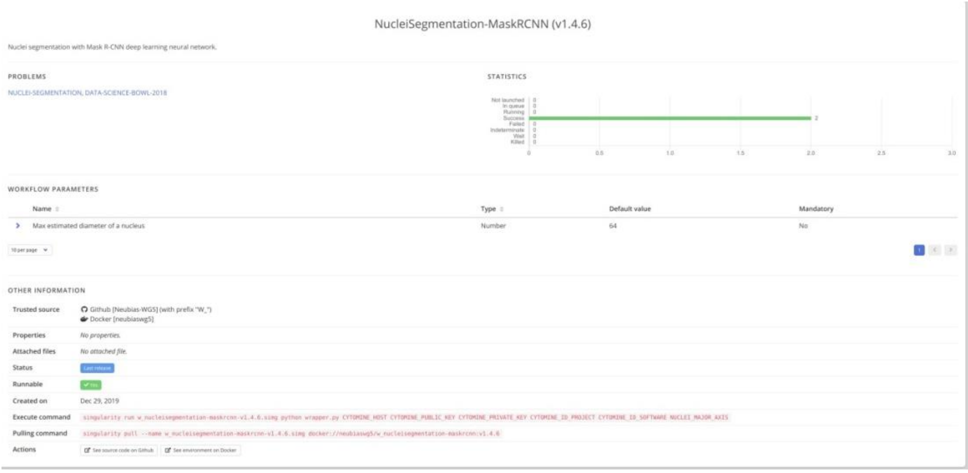

BIAFLOWS workflows page. Details of Mask R-CNN workflow with direct access to versioned source code on GitHub.

Section 2. Installing and populating BIAFLOWS locally

It is possible to install BIAFLOWS on a local server or a desktop computer. This might be useful to manage and analyse images locally or organize challenges. The procedure is described below and should take less than 30 minutes (UNIX-like based system recommended).

Installing a local instance of BIAFLOWS

The procedure described in this section is for Linux Ubuntu but it should be possible to install BIAFLOWS on other platforms (not tested). Some specific details related to deployment on Mac OS can be found online:
https://doc.uliege.cytomine.org/display/PubOp/Install+Cytomine+on+MacOS

1/ Installation requirements
BIAFLOWS runs in Docker containers, the only requirement is to install Docker.
Check the official Docker documentation to install Docker for Ubuntu:
https://docs.docker.com/install/linux/docker-ce/ubuntu/

Choose *Install using the repository*, set up the repository and install Docker CE.

2/ Retrieve BIAFLOWS installation files by typing the following commands in a terminal

```
mkdir Biaflows/
cd Biaflows/
git clone https://github.com/Neubias-WG5/Biaflows-bootstrap.git
cd Biaflows-bootstrap
```

3/ Configure the local instance

Edit **configuration.sh** and, if necessary, update URLs (CORE_URL, IMS_URL, UPLOAD_URL). Make sure to use URLs that are not already used by other applications (avoid **localhost**) to prevent conflicts. In **/etc/hosts** of the host machine, add the following lines, adapting them accordingly to chosen XXX_URL in **configuration.sh**.

127.0.0.1 biaflows

127.0.0.1 biaflows-ims

127.0.0.1 biaflows-upload

127.0.0.1 rabbitmq

If needed, update data path variables (**IMS_STORAGE_PATH**…) in **configuration.sh**. All data paths must be valid and mappable in the Docker engine. If they don't exist, create the directories (**mkdir**) corresponding to the following variables:

A reference to these URLs and paths is provided here:
https://doc.uliege.cytomine.org/display/PubOp/Cytomine+configuration+reference

Configure **BIAFLOWS_WORKFLOWS_METRICS** to *true* or *false* depending if you want to perform benchmarking or not on this instance: ground truth annotations are then required for all images. Set this flag to false if you plan to manage / process local images only.

4/ Initialize the deployment
Run the installation script: sudo bash init.sh

5/ Deploy the local instance
Run the generated deployment script: sudo bash start.sh

6/ Check the running instance

When startup is finished, check that the application is running in your browser at the URL specified by **CORE_URL** (default: http://biaflows).
Three accounts with different access rights are automatically created (username: admin; password: admin; username: guest; password: guest; username: neubias; password: neubias). Passwords should be updated from the Account page (top right).

7/ Install sample Problems (images and ground-truth data)
After BIAFLOWS is successfully installed locally, the local instance is empty. All projects available in BIAFLOWS online instance can be imported to the local instance. For this, get the public and private keys of the admin account (Account page), then run:

```
cd Biaflows-bootstrap
sudo bash ./inject_demo_data.sh ADMIN_PUBLIC_KEY ADMIN_PRIVATE_KEY
```

where **ADMIN_PUBLIC_KEY** and **ADMIN_PRIVATE_KEY** have been substituted with their respective values.

The script starts to download projects and import them in your local BIAFLOWS.
The list of imported projects can be tweaked by editing the file
Biaflows-bootstrap/configs/project_migrator/projects.txt.

The whole data injection procedure can take several minutes, depending on your Internet connection and the number of projects being imported.

Creating a new Problem (project) in a local BIAFLOWS instance

To create a new problem, connect as regular user or admin.

1/ Go to the **Problems** tab

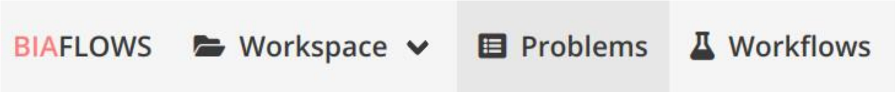

2/ Click **New Problem**

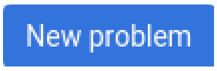

3/ Choose a meaningful problem name and save

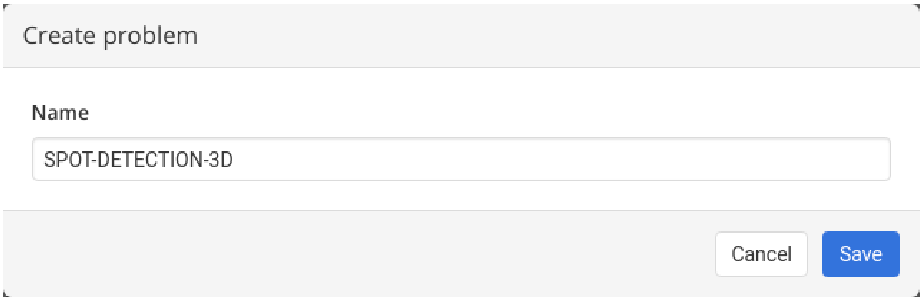

4/ The problem is ready to be configured, the following configuration is recommended

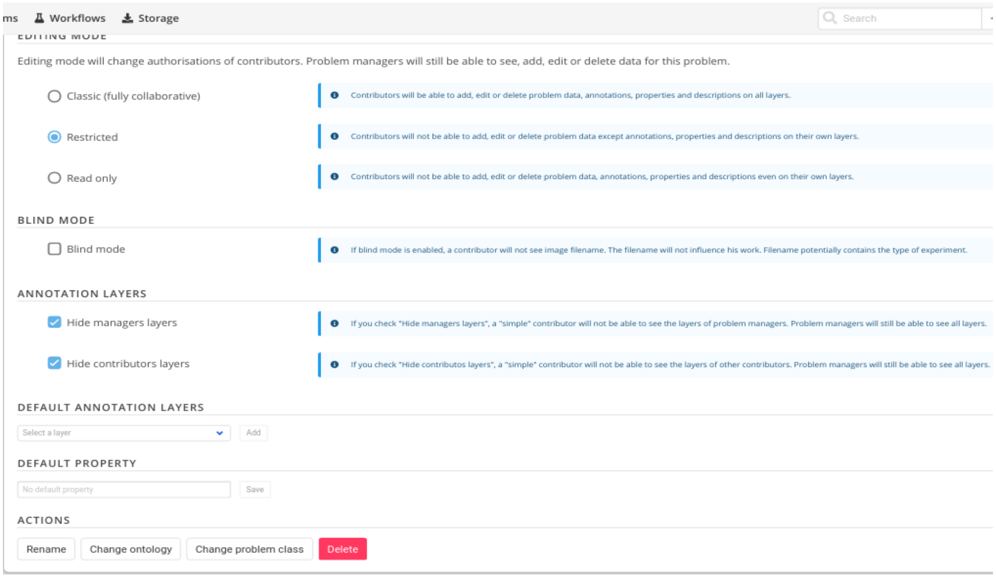

5/ Assign your problem to a problem class (see Section 4 **Problem Class, Ground truth annotations and reported metrics**) by clicking on **Change problem class**. The problem class specifies the format of ground truth annotations (and workflow outputs), as well as the associated benchmark metrics to be computed (if benchmark is enabled).

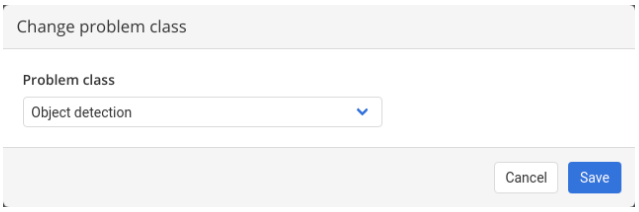

6/ Configure project members. If you work alone, you can leave contributors and project managers to default user. This can be done from the “Members” tab in the problem configuration.

7/ The problem can be fully configured to display or hide panels / tabs / tools in the user interface. This is achieved from the **Custom UI** tab in the problem configuration.

8/ A description of the problem can optionally be added from the **Information** (left sidebar). The description is displayed in **Problems** list.

Uploading images to a local BIAFLOWS instance

To upload new images, connect as regular user or admin.

Supported formats

- **2D images**: 8-bit/16-bit TIFF (or OME-TIFF files)
- **Multi-dimensional images (Z, C, T)**: single file 8-bit/16-bit OME-TIFF

Note: The text string **_lbl** should not be used in image names since it is a reserved string for ground truth annotation images.

1/ Go to **Storage** section

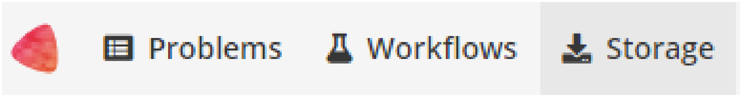

2/ Select the **Problem** to which the images should be associated with (**Link with problem**)

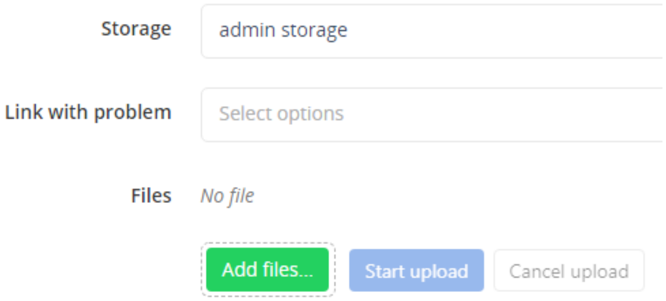

__Note__: If a problem is not in the list, make sure you are a member for this problem

3/ Click on **Add files…** and select the files from the file browser

4/ Start upload with **Start upload** and wait until completion

The status can be:

- **DEPLOYED/CONVERTED**: The image is correctly imported to BIAFLOWS
- **ERROR FORMAT**: The file format is not supported
- **ERROR EXTRACTION**: Something went wrong during metadata extraction
- **ERROR CONVERSION**: Something went wrong during the conversion of the image into the BIAFLOWS internal image format
- **ERROR DEPLOYMENT**: Something went wrong during the communication with BIAFLOWS API. It can be due to access rights, or other unexpected error

Note: Images uploaded to storage can also be associated to a Problem after upload (Problem: **Add image**). This can be useful to associate the same image to several Problems.

Uploading ground truth annotations to an existing BIAFLOWS problem

If you plan to perform benchmarking, ground truth annotations should also be uploaded and associated to every image of a problem. The format of these annotations depends on the associated problem class (see Section 4 **Problem Class**, **ground truth annotations and reported metrics**).

Image annotations (e.g. binary masks) should be uploaded as 16-bit TIFF (or OME-TIFF) for 2D images and as single file 16-bit OME-TIFF for multidimensional (C,Z,T) images. They should be uploaded by following the procedure described in the previous section and by setting the same name as their corresponding image + **_lbl** suffix (e.g. **AnImage.ome.tif** and **AnImage_lbl.ome.tif**).

Other required annotations (e.g. SWC, division text file) should be added to the images as attached files. To do so, expand the image (blue arrow) in the list and click on **Add** next to **Attached files**. These can also be added programmatically using our Python client.

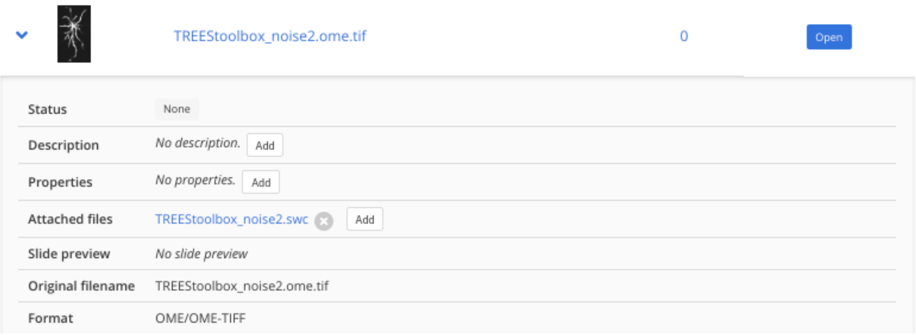

Adding existing workflows from trusted sources to a local BIAFLOWS instance

It is possible to integrate existing BIAFLOWS workflows to any BIAFLOWS instance. This operation requires configuring an external trusted source made of:

1. A source code registry (typically a GitHub user space)
2. An execution environment registry (typically a DockerHub user space)

If your workflow repositories are mixed with other repositories in your user space, you can specify a prefix to distinguish workflow repositories. For instance, all bioimage analysis workflows developed by NEUBIAS are prefixed by **W_** and available from this user space: https://github.com/Neubias-WG5.

Some information regarding trusted sources is given below.

To manage trusted sources, you need to be administrator.

1/ Connect as administrator by clicking Open admin session:

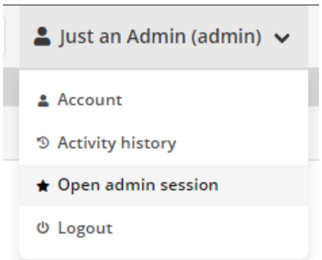

2/ In the administration page, go to **Trusted sources** tab and click **Add trusted source**

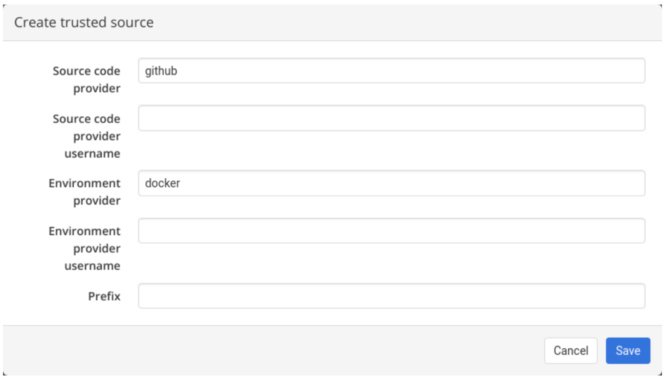

3/ Fill the form and **Save**

For instance, to add NEUBIAS curated set of workflows, the trusted source has to be configured as follows:

- **Source code provider:** github
- **Source code provider username:** Neubias-WG5
- **Environment provider:** docker
- **Environment provider username:** neubiaswg5
- **Prefix:** W_

4/ Trusted sources are periodically checked (about every 10 minutes) to automatically add new versions of existing workflows or new workflows, but you can also click on **Refresh** to trigger the check.

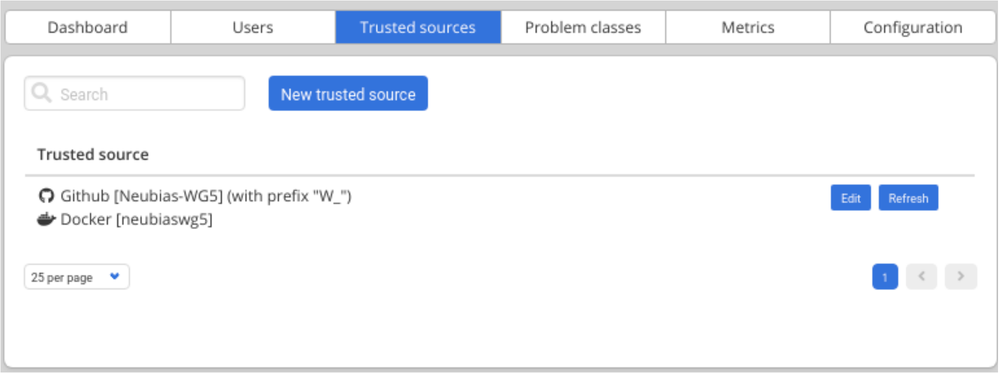

5/ Once a workflow is imported, it has to be linked to a BIAFLOWS **Problem**. This can be performed in the Configuration panel of the **Problem** (**Workflows** tab) by toggling **Enable** for that workflow as illustrated below:

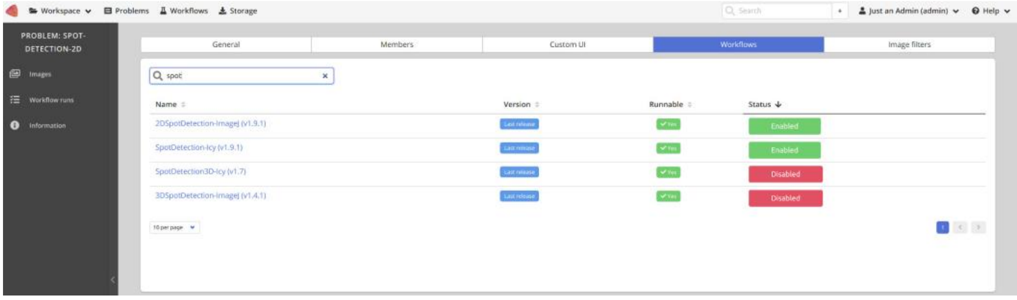

Section 3. Creating a BIA workflow and adding it to a BIAFLOWS instance

Introduction

BIAFLOWS workflows are *Docker images* encapsulating a complete execution environment together with a workflow addressing a BIA Problem. These *Docker images* can be compiled automatically online. BIAFLOWS instances automatically fetch new workflows and make them available from the user interface. Sample workflows running in ImageJ (macros and scripts), ICY, CellProfiler, ilastik, Vaa3D, Python, Octave and Jupyter notebooks can be found in this GitHub repository: https://github.com/neubias-wg5. The procedure to package a workflow and add it to a BIAFLOWS instance is described in this section. Users wishing to get help can write to https://forum.image.sc forum or contact biaflows@neubias.org.

BIA workflow requirements

BIAFLOWS workflows must:

- Run headless from command line
- Take an input folder of 8 bit/16 bit TIFF (2D) or single file OME-TIFF (C,Z,T) images
- Expose functional parameters and parse them from command line call
- Export results to an output folder in a format specified for the Problem Class (see **Problem Class, ground truth annotations and reported metrics**).

The workflow and its software execution environment are fully defined from a set of 4 files:

- A DockerFile configuring software execution environment (OS, libraries, software...)
- The workflow executable or, more commonly, a script running on a BIA platform
- A Python script (wrapper.py), sequencing operations (*Docker image* entry point)
- A descriptor (descriptor.json) specifying workflow parameters and default values.

Note: A wizard is now available to add new workflows. This tool is useful if you plan to add a new workflow from an existing combination of BIA problem class and target BIA platform (e.g. Object segmentation 2D + ImageJ), acting as a template.
For details, please refer to: https://github.com/Neubias-WG5/biaflows-workflow-utilities.

Step 1. Create a workflow GitHub repository

Create a workflow repository in a GitHub source trusted by the BIAFLOWS instance you plan to add the workflow to. The names of workflow repositories should start by a fixed prefix (**W_** recommended since it is the convention used by BIAFLOWS online instance) and contain no space.

Step 2. Add the 4 required files to the workflow repository

It is recommended to reuse existing files from similar workflow repositories in https://github.com/Neubias-WG5. For this, follow these guidelines:

- A descriptor from the **Problem Class** you target (e.g. Object Segmentation)
- A DockerFile configuring the BIA platform you target (e.g ImageJ)
- A wrapper script from the **Problem Class** and the **workflow type** you target.

Note: The flag **is_2d** specifies if the images from the Problem hold two spatial dimensions (three spatial dimensions if set to false).

The following workflow types have already been tested and are available from https://github.com/Neubias-WG5: ImageJ / FIJI macro, ImageJ Python script, ICY protocol, CellProfiler pipeline, Octave script, ilastik pipeline, Vaa3D plugin, Python 2.X or 3.X script based on Scikit-learn or Keras/Pytorch.

Step 3. Update the following sections of the **Descriptor**

Workflow and associated Docker image names

```
{
    "name": "NucleiTracking-ImageJ",
    "container-image": {
        "image": "neubiaswg5/w_nucleitracking-imagej",
        "type": "singularity"
}
```

Update *name* to match GitHub workflow repository name (without prefix)
Update *image* to match the name of your workflow GitHub repository (lower case only)

Command line call of the Docker image

```
"description": "Track nuclei in a time series by doing 3D segmentation.",
"command-line": "python wrapper.py CYTOMINE_HOST CYTOMINE_PUBLIC_KEY
 CYTOMINE_PRIVATE_KEY CYTOMINE_ID_PROJECT CYTOMINE_ID_SOFTWARE
IJ_RADIUS IJ_THRESHOLD IJ_ERODE_RADIUS ",
```

Description: Update workflow description
Command-line: Update parameter list (here last 3 arguments)

Workflow parameter sections

```
{
    "id": "ij_gauss_radius",
    "value-key": "@ID",
    "command-line-flag": "--@id",
    "name": "Radius",
    "description": "Radius for the Gaussian filter",
    "type": "Number",
    "default-value": 3,
    "optional": true
}
```

Update / add as many parameter sections as required to match the parameter list from command line call.

id: should match parameter name in command line call (lower case)
name: name that will appear in BIAFLOWS user interface (parameter dialog box)
description: context help in BIAFLOWS user interface (parameter dialog box)
type: “String” or “Number”
default-value: the default value in BIAFLOWS user interface (parameter dialog box).

Step 4. Update DockerFile

Update the line copying the workflow from the GitHub repository to the workflow Docker image, for instance:

ADD NucleiTracking.ijm /fiji/macros/macro.ijm

If necessary, append commands to install additional required libraries/plugins to the execution environment.

Step 5. Update wrapper script

Update workflow command line call in wrapper.py.

```
command = "/usr/bin/xvfb-run ./ImageJ-linux64 -macro macro.ijm
input={}, output={}, ij_radius={}, ij_threshold={}, ij_erode_radius={}\" -batch"
.format(in_path, out_path, nj.parameters.ij_radius,nj.parameters.ij_threshold, nj.parameters.ij_erode_radius)
```

Update/add parameters to match parameters defined in JSON descriptor (Step 2).

Step 6. Adapt your workflow script

Adapt your workflow script to fulfil workflow requirements and parse parameters from command line. For instance for an ImageJ macro:

```
for(i=0; i<parts.length; i++) {
    nameAndValue = split(parts[i], "=");
    if (indexOf(nameAndValue[0], "input")>-1) inputDir=nameAndValue[1];
    if (indexOf(nameAndValue[0], "output")>-1) outputDir=nameAndValue[1];
    if (indexOf(nameAndValue[0], "gauss_rad")>-1) GaussRad=nameAndValue[1];
    if (indexOf(nameAndValue[0], "threshold")>-1) Thr=nameAndValue[1];
    if (indexOf(nameAndValue[0], "open_rad")>-1) OpenRad=nameAndValue[1];

}

images = getFileList(inputDir);
for(i=0; i<images.length; i++)
{
… DO SOMETHING..
}
```

Step 7. Create Docker image in DockerHub

Sign in to DockerHub and create a new public repository. The repository name must match the container-image name used in Step 3.

Step 8. Link repository to workflow GitHub repository and configure workflow *Docker image* automated build according to the following example:

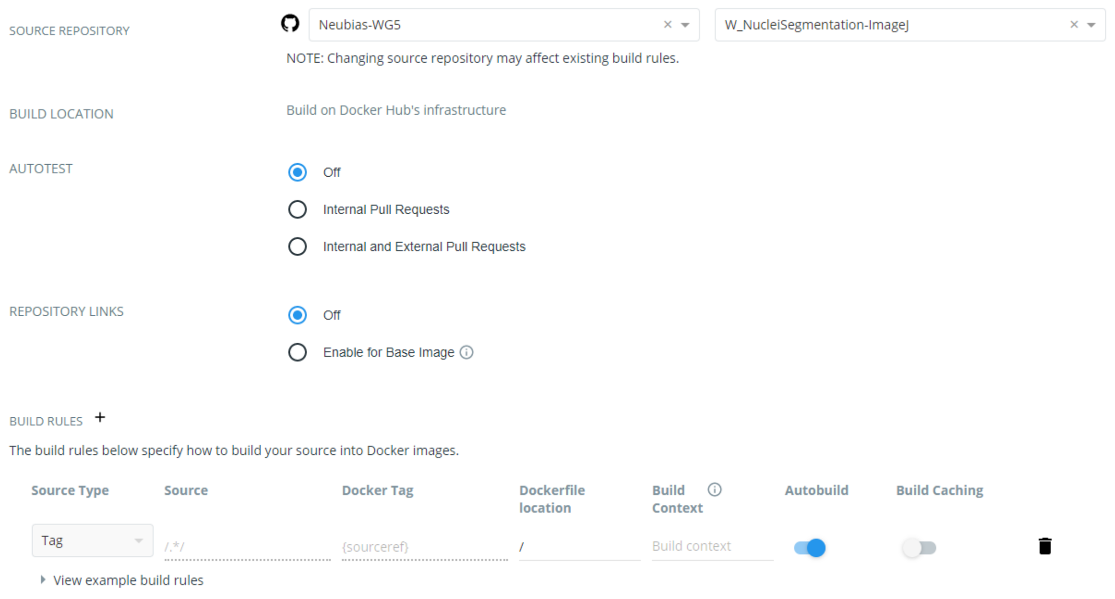

Step 9. Trigger a workflow release

Trigger a release from GitHub workflow repository with version tag such as 0.1, 0.2, 1.0...

Step 10. Workflow Docker image build

Check from DockerHub that the workflow *Docker image* has built successfully. If not, parse the log and fix issues by modifying DockerFile and retriggering a new release.

Step 11. Add workflow to BIAFLOWS problem

Once the *Docker image* is built, a BIAFLOWS instance fetches the image from the trusted source and make it available (possibly after up to 5/10 minutes). Sign in as administrator to BIAFLOWS and browse to the **Problem** you want to add the workflow to. Then, click on the **Configuration** icon (bottom left of the side bar).

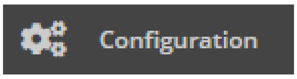

Search for the workflow (recently added workflows are on top of the list) and enable it. Older workflow versions can be disabled if this is an update to an existing workflow.

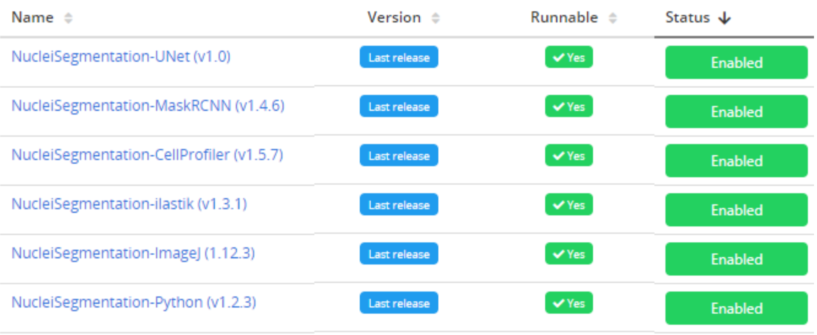

Name	Version	Runnable	Status
NucleiSegmentation-UNet (v1.0)	Last release	Yes	Enabled
NucleiSegmentation-MaskRCNN (v1.4.6)	Last release	Yes	Enabled
NucleiSegmentation-CellProfiler (v1.5.7)	Last release	Yes	Enabled
NucleiSegmentation-ilastik (v1.3.1)	Last release	Yes	Enabled
NucleiSegmentation-ImageJ (1.12.3)	Last release	Yes	Enabled
NucleiSegmentation-Python (v1.2.3)	Last release	Yes	Enabled

Step 12. Run the workflow

Test the workflow by running it from BIAFLOWS / **Workflow runs** (requires execution rights).

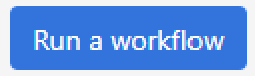

If execution fails, read the execution log, update the code and trigger a new release.

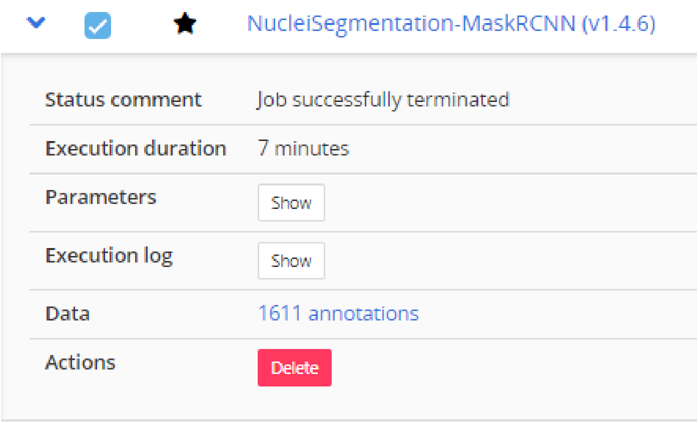

Detailed Developer guide

This section provides some more details on BIAFLOWS workflows and details how to compile and debug BIAFLOWS workflows Docker image locally and add them to an existing BIAFLOWS instance.

Details on Python wrapper script and JSON descriptor

The sequence of operations commonly performed by BIAFLOWS Python wrapper scripts is detailed in following table. All workflows provided in BIAFLOWS repository follow this template. A complete reference to BIAFLOWS workflows JSON descriptor can be found online: https://doc.uliege.cytomine.org/display/ALGODOC/Software+JSON+descriptor+reference (

Phase	Actions	Notes
**Initialization***	Connect to BIAFLOWS<br>Retrieve Problem Class<br>Retrieve job parameters	
**Prepare_data***	Create empty in_folder, out_folder, gt_folder, tmp_folder<br>Download all images without _lbl suffix to in_folder<br>Download all images with _lbl suffix to gt_folder<br>Download all image file attachments to gt_folder	Folders are created in user home folder (gt = ground truth). File names: annotation files must have the same name as input images + *_attached*
**Workflow call**	Call workflow from command line and passing in_folder, out_folder and parameters	The images from in_folder are sequentially processed, the results are stored in out_folder
**Upload_data***	Parse images from out_folder (typically binary masks) and for each image/slice create annotations (polygon or point) and export them to BIAFLOWS	(1) Plain objects: extract connected particles (2D/3D) from mask, create polygon contours (slice by slice) and set contour ID (color LUT) to mask object ID<br>(2) Points: Find non null pixels/voxels. Create point annotation at this position<br>(3) Skeletons: Project mask (fully or by block), dilate, find contour around skeleton
**Upload_metrics***	For each input file: call ComputeMetrics passing pairs of out_file(s) / gt_file(s), problem class (string) and optional metric parameters. Export metrics keys/values to benchmark database	gt_file: same name as out_file<br>If an attached file is expected (e.g. division text file), it is assumed at the same location as out_file / gt_file and with the same name as the image + *_attached*

Typical steps of a BIAFLOWS Python wrapper script. Phases with * can be skipped (depends on the flags passed to workflow container). For instance, for the local processing (no BIAFLOWS server) all steps are skipped while for a local BIAFLOWS instance upload_metrics may be skipped if the images to be processed are not annotated.

Installing software required for development (only once)

As workflows run inside a Docker container and since their Python wrapper script interacts with a BIAFLOWS instance, it is required to install Docker and Python 3 on your local machine. Our Python client is also required for development. Docker installation instructions can be found here:

For Linux:
https://www.digitalocean.com/community/tutorials/how-to-install-and-use-docker-on-ubuntu-18-04

For Windows:
https://docs.docker.com/docker-for-windows/install/#install-docker-for-windows-desktop-app

Python 3 and Cytomine Python client instructions can be found here:
https://doc.uliege.cytomine.org/display/ALGODOC/Data+access+using+Python+client

In the following steps, we will use the workflow “NucleiSegmentation-ImageJ” as reference:
https://github.com/Neubias-WG5/W_NucleiSegmentation-ImageJ

Step 1. Uploading a new workflow descriptor to BIAFLOWS

Workflows have first to be described through a JSON descriptor, e.g.:
https://github.com/Neubias-WG5/W_NucleiSegmentation-ImageJ/blob/master/descriptor.json

Currently, some sections have to be customized manually, and some conventions must be respected to allow automatic parsing by BIAFLOWS. We recommend using https://github.com/Neubias-WG5/W_Template/blob/master/descriptor.json as template for your JSON descriptor.

Choose a workflow name that does not contain space. The description field (supporting restricted HTML) should be filled to document the workflow and it will be displayed from BIAFLOWS UI.

As inputs (workflow parameters), the five parameters (**CYTOMINE_HOST CYTOMINE_PUBLIC_KEY, CYTOMINE_PRIVATE_KEY, CYTOMINE_ID_PROJECT, CYTOMINE_ID_SOFTWARE**) are mandatory.

The fields associated to workflow parameters are described here:

- id: the parameter name (e.g : “ij_radius”)
- value-key: a reference for the parameter in the command line. Keep “@ID”, which is a shorthand meaning “replace by the parameter id, in uppercase”. In our example, it will be replaced at parsing time by “IJ_RADIUS”
- command-line-flag: At execution time, the value-key in the command line will be replaced by the command-line-flag followed by the parameter value. Keep “--@id”. In our example, it will be replaced in the command line by “--ij_radius”.
- name: a human readable name displayed in BIAFLOWS
- type: Number, String, Boolean
- optional: set to true only if the workflow execution is not influenced by the presence or the absence of the parameter (e.g a “verbose” parameter). Workflow parameters having an influence on the results should never be optional.
- default-value: the default value of the parameter (in BIAFLOWS interface).

Do not forget to update the parameter value keys in the command line.
For instance, for workflow parameters ij_radius and ij_threshold:

python wrapper.py CYTOMINE_HOST … IJ_RADIUS IJ_THRESHOLD

To make a workflow available from a BIAFLOWS instance, it is currently required to publish its descriptor using Cytomine Python client. This can be performed by running the following Python code inside the folder holding the JSON descriptor you have created:

```
from cytomine import Cytomine
from cytomine.utilities.descriptor_reader import read_descriptor
with Cytomine(host, public_key, private_key) as c:
        read_descriptor("descriptor.json")
```

host is the url of your BIAFLOWS server, e.g. https://biaflows.neubias.org
public_key and ***private_key*** can be found from user Account page (section API KEYS)

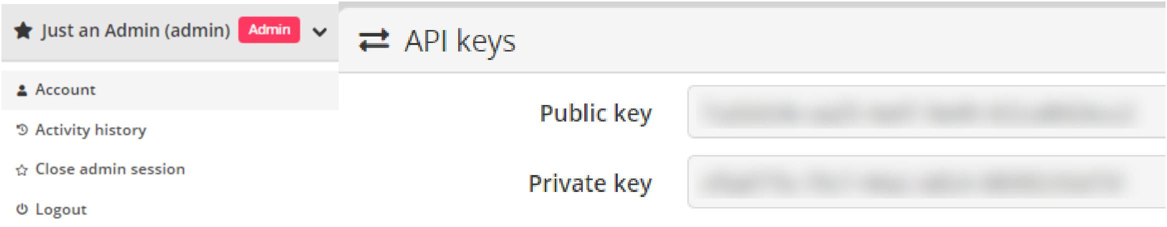

Step 2. Linking a new workflow to a BIAFLOWS project

- From *Problems*, select the problem to which you want to add the workflow
- Go to *Problems* > *Configuration* > *Workflows* and enable the workflow

For now, as the workflow has been added manually, it will be referenced as **Not Runnable** and no version information will be provided from the UI.

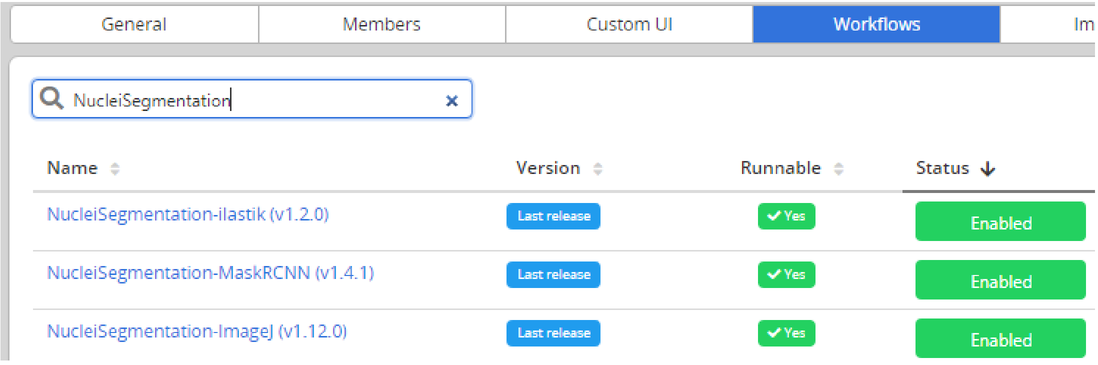

Next, Go to *Projects* > *Configuration* and make sure that Jobs tab is activated (green)

Step 3. Creating the DockerFile

Docker files specify the execution environment. They typically start by creating (FROM) a layer from an existing Docker image with basic operating system. Then they execute commands (RUN) to install specific software and libraries, and copy (ADD) files (e.g. the Python wrapper script and workflow script) into the execution environment the workflow will be called from. Finally, the ENTRYPOINT is set to the wrapper script.

A sample DockerFile is available here:
https://github.com/Neubias-WG5/W_NucleiSegmentation-ImageJ/blob/master/Dockerfile

If you do not know how to configure the DockerFile, it is recommended to adapt the DockerFile from an existing BIAFLOWS workflow using the same target software (e.g. an ImageJ macro).

Note: If you create a DockerFile from scratch, always use the most accurate tag when referring to an existing Docker image (e.g. prefer python:3.6.9-stretch over python:3.6). If the tag is not accurate, the underlying Docker image could change over time, heavily impairing reproducibility!

Step 4. Creating the wrapper script

It is recommended to adapt a wrapper script: 1) from same problem class, 2) processing image of same dimensionality (e.g. 3D), and 3) matching the software you are planning to use (e.g. ImageJ macro). In this case, only the workflow call (command line) needs to be adapted. A sample wrapper script is available here:
https://github.com/Neubias-WG5/W_NucleiSegmentation-ImageJ/blob/master/wrapper.py

Note: The flag **is_2d** should be used to specify if the images are strictly 2d or multidimensional.

Step 5. Building the workflow image, running it in a local container and debugging

A new workflow can be directly pushed to GitHub and be built in DockerHub, but it is preferable to test it locally beforehand. For this, it is required to build and run the Docker image locally:

Building the container (you need at least around 5GB disk space for this operation)

From a directory where you gathered the 4 files required to describe the workflow:

```
cd ~/Documents/Code/NEUBIAS/W_NucleiSegmentation-ImageJ$
sudo docker build -t seg2d .
```

Here seg2d is the name of the Docker image to build locally.

Running the Docker image:

```
sudo docker run -it seg2d --host host --public_key public_key --private_key private_key --software_id software_id --project_id project_id --ij_threshold 15 --ij_radius 4
```

The list of command line parameters should exactly match the parameters defined in the JSON descriptor file. BIAFLOWS instance URL and credentials should also be filled, as well as valid **workflow_id** (using **--software_id**) and **problem_id** (using **--project_id**).

These IDs can be retrieved from the URL bar while respectively clicking on a problem (from BIAFLOWS **Problems** tab) and on a workflow (from BIAFLOWS **Workflows** tab):

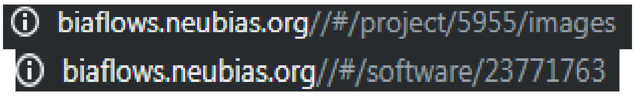

In this example, workflow_id=23771763 and problem_id=5955.

If a workflow fails at execution this is reported in **Workflow runs** section. Some **Execution log** information can be downloaded by expanding a workflow run from the blue arrow:

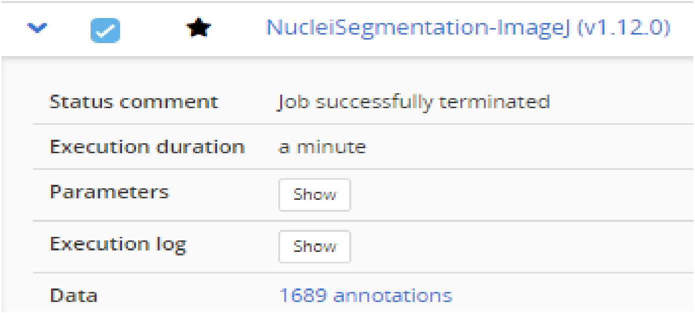

In this case, no associated benchmark metric is associated to this run. There is hence no risk that this would be left unnoticed by the user. For debugging, Docker can be run with an interactive session:

```
sudo docker run --entrypoint bash -it seg2d
```

If needed, it is also possible to launch the Docker with X enabled, e.g. to debug an ImageJ macro more easily:

```
xhost + sudo docker run --entrypoint bash -v /home/yourusername/tmp/test:/data -e DISPLAY=$DISPLAY -v /tmp/.X11-unix:/tmp/.X11-unix -it seg2d
```

If you want to access local images without having to download them each time from BIAFLOWS, you can also attach a local folder to a folder inside the Docker container (-v option), for instance:

```
sudo docker run --entrypoint bash -v /home/yourusername/tmp/test:/data -it seg2d
```

Some other useful Docker commands

Check if an image is running: ps -a
Remove a running container: sudo docker rm CONTAINER_ID
Remove all running containers: sudo docker rm $(sudo docker ps -a -q)
Download a specific container sudo docker pull neubiaswg5/fiji-base:latest

Note: To download a recently updated workflow image, it is necessary to first remove older versions manually.

Step 6. Publishing a workflow with version control

Once your workflow is running properly, you can officially publish it with version control.

To allow automatic import to BIAFLOWS, the set of files previously described should be stored in a GitHub repository (linked to DockerHub) from an account trusted by the target BIAFLOWS instance.

The Github repository name must be given by:

Github repo name = {prefix}{workflow_name}

where:

- {prefix} is an optional prefix for the trusted source (see **Installing and populating BIAFLOWS locally**)
- {workflow_name} is the name of the workflow as given in the "name" field in the JSON descriptor (see Step 1).

For instance, for a trusted source with a prefix **W_**: **W_NucleiSegmentation-ImageJ**.

Adding/editing trusted sources is performed from Admin / Trusted sources (**Installing and populating BIAFLOWS locally**):

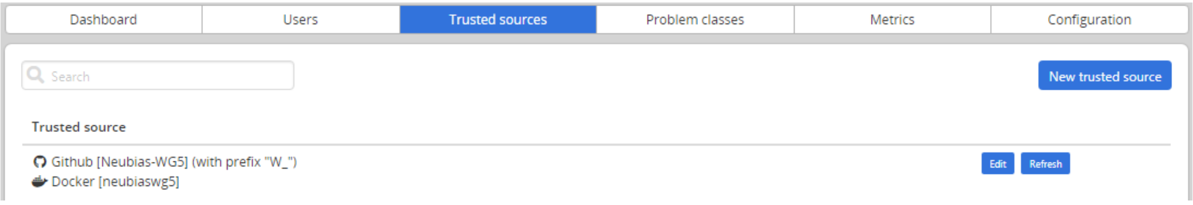

Step 7. Linking a GitHub repository to DockerHub (only once)

We assume that you created a trusted GitHub organization (e.g. **neubias-wg5**) and a workflow repository holding the 4 workflow files. It is now required to link DockerHub to GitHub. Fortunately, this operation has to be performed only once for a given GitHub organization:

1. Create an account on DockerHub : https://hub.docker.com/ and login
2. Create an automatic build by linking Docker account to GitHub organization account
3. On DockerHub website, click on Create > Create Automated Build

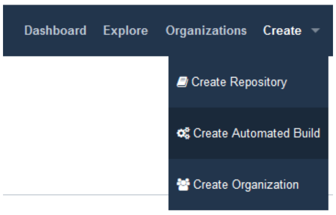

4. In **Linked Accounts,** click on **Link Github**

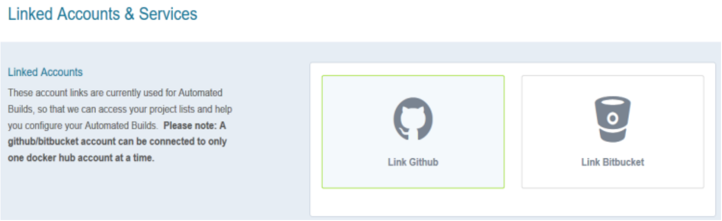

5. Click **Select**
6. Ensure that Organization access (e.g. **Neubias-WG5)** is selected (green check mark) and click on **Authorize docker**
7. Enter your GitHub password to enable access

Step 8. Associating a new workflow repository to DockerHub

Once your GitHub organization account and DockerHub are linked, it is possible to create an automated build procedure for each workflow. This procedure will build a workflow Docker image each time a new release is triggered from a GitHub workflow repository. This image is automatically downloaded by the BIAFLOWS instance and the new workflow version will be available for the target problem.

To do so, from DockerHub:

1. Click on **Create > Create Repository+**
2. In build settings click on GitHub icon
3. Select organization (e.g. **neubiaswg5**) and workflow Github repository (e.g. **W_NucleiSegmentation-ImageJ**) at the bottom of the page
4. Choose the Docker registry repository name. In practice, keep the same as GitHub repository (DockerHub will convert uppercase letters into lowercase).
5. Enter a short description (less than 100 characters) and click **Create**
6. Click on ***Click here to customize the build settings*** and configure as in figure below
7. Click on Save

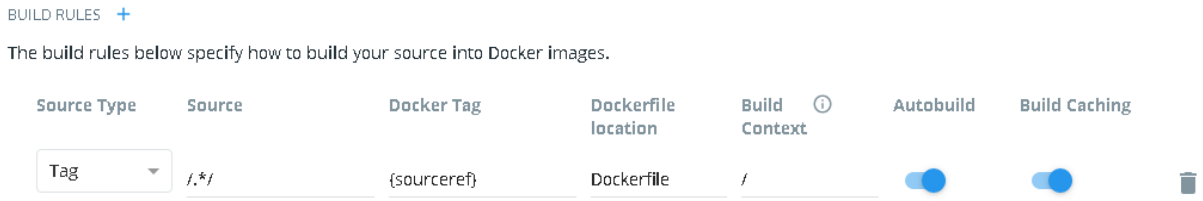

The DockerHub repository name must be reflected in the JSON descriptor:

image = {dockerhub_organization}/{github_repo_name.toLowerCase()}

For example, in the JSON descriptor:

container-image:
{
    image: "neubiaswg5/w_nucleisegmentation-imagej",
    type: "singularity"
},

Step 9. Creating a versioned release on GitHub

To create versioned releases of the workflow, go to GitHub and draft a new release (see https://goo.gl/bFz66N). This will add a new tag to the last commit. As we configured automatic build in previous step, a new Docker image will be built and published with the same tag. BIAFLOWS instances trusting this GitHub / DockerHub repository will now automatically fetch and make this new version available from the UI (this may take up to 5/10 minutes).

Section 4. Problem class, ground truth annotations and reported metrics

To perform benchmarking, ground truth annotations should be encoded in a format that is specific to the associated problem class. BIA workflows are also expected to output results in the same format. Currently 9 problem classes are supported in BIAFLOWS and their respective annotation formats and computed benchmark metrics are described below.
Each problem class has a long name (explicit) and short name, for instance Object Segmentation (ObjSeg). The same hold for metrics, for instance DICE (DC). See section 6 **Benchmark Metrics** for metrics description.

Problem class: Object Segmentation (ObjSeg)

Task: Delineate objects or isolated regions

Object Encoding: 2D/3D label masks with foreground > 0 (one unique ID per object), background = 0

Reported metrics: DICE (DC), AVERAGE_HAUSDORFF_DISTANCE (AHD), computed by VISCERAL executable (archived here), Fraction overlap (FOVL) computed by custom Python code, Mean Average Precision computed by Data Science Bowl 2018 Python code.

Problem class: Spot / object counting (SptCnt)

Task: Estimate the number of objects

Object Encoding: 2D/3D binary masks, exactly 1 spot/object per non null pixel

Reported metrics: RELATIVE_ERROR_COUNT (REC), computed by custom Python code.

Problem class: Spot / object detection (ObjDet)

Task: Detect objects in an image (e.g. nucleus)

Object Encoding: 2D/3D binary masks, exactly 1 object per non null pixel

Reported metrics: CONFUSION_MATRIX (TP, FN, FP), F1_SCORE (F1), PRECISION (PR), RECALL (RE), Distance RMSE (RMSE), computed by Particle Tracking Challenge metric Java code (particle matching only, archived here in bin / DetectionPerformance.jar)

Problem class: Pixel/Voxel Classification (PixCla)

Task: Estimate pixels class

Object Encoding: 2D/3D class masks, gray level encodes pixel/voxel class, background = 0

Reported metrics: F1_SCORE (F1), ACCURACY (ACC), PRECISION (PR), RECALL (RE), computed by custom Python code

Problem class: Filament Tree Tracing (TreTrc)

Task: Estimate the medial axis of a connected filament tree network (one per image)

Object Encoding: SWC file

Reported metrics:
UNMATCHED_VOXEL_RATE (UVR), computed by custom Python code

NetMets metrics: Geometric False Negative rate (FNR), Geometric False Positive rate (FPR) computed by NetMets Python code

Metrics parameters:
GATING_DIST (UVR): Maximum distance between skeleton voxels in reference and prediction skeletons to be considered as matched (default = 5 pix)
Sigma (NetMets): tolerance in centreline position (default: 5 pix)

Problem class: Filament Networks Tracing (LooTrc)

Task: Estimate the medial axis of one or several connected filament network(s)

Object Encoding: 2D/3D skeleton binary masks with skeleton pixels > 0, background = 0

Reported metrics:
UNMATCHED_VOXEL_RATE (UVR), computed by custom Python code.
NetMets metrics: Geometric False Negative rate (FNR), Geometric False Positive rate (FPR) computed by NetMets Python code

Metrics parameters:
GATING_DIST (UVR): Maximum distance between skeleton voxels in reference and prediction skeletons to be considered as matched (default = 5 pix)
Sigma (NetMets): tolerance in centreline position (default: 5 pix)
Skeleton sampling distance (NetMets): skeletons are sampled to be converted to SWC models. (default: 3 voxels, default Z Ratio: 1)

Problem class: Landmark Detection (LndDet)

Task: Estimate the position of specific feature points

Object Encoding: 2D/3D class masks, exactly 1 landmark per non null pixel, gray level encodes landmark class (1 to N, N is the number of landmarks)

Reported metrics: Number of reference / predicted landmarks (NREF, NPRED), Mean distance from predicted landmarks to closest reference landmarks with same class (MRE). All metrics computed by custom Python code

Problem class: Particle Tracking (PrtTrk)

Task: Estimate the tracks followed by particles (no division)

Object Encoding: 2D/3D label masks, exactly 1 particle per non-null pixel, gray level encodes particle track ID

Reported metrics: Normalized pairing score alpha (NPSA), Full normalized pairing score beta (FNPSB), Number of reference tracks (NRT), Number of candidate tracks (NCT), Jaccard Similarity Tracks (JST), Number of paired tracks (NPT), Number of missed tracks (NMT), Number of spurious tracks (NST), Number of reference detections (NRD), Number of candidate detections (NCD), Jaccard similarity detections (JSD), Number of paired detections (NPD), number of missed detections (NMD), Number of spurious detections (NSD)

All metrics computed by Particle Tracking Challenge Java code (archived here)

Metrics parameters: GATING_DIST (default = 5, maximum distance between particle detections in reference / prediction tracks to be considered as matching)

Problem class: Object Tracking (ObjTrk)

Task: Estimate object tracks and segmentation masks (with possible divisions)

Encoding: 2D/3D TIFF label masks, gray level encodes object ID + division text file (see Cell Tracking Challenge format)

Reported metrics: Segmentation measure (SEG), Tracking measure (TRA). All computed from Cell Tracking Challenge metric command line executables (archived here and here)

Section 5. Additional features

This section describes additional tools, different ways to interact with a BIAFLOWS server, and content migration / importation.

Using BIAFLOWS as an image source for a Jupyter notebook

The procedure is self-documented online, press launch binder from this GitHub repository: https://github.com/Neubias-WG5/biaflows_jupyter_minimal

Importing existing datasets from a BIAFLOWS instance

Content migration from an existing instance (e.g. BIAFLOWS online instance) to a local instance is possible. To migrate data, we developed tools that rely on BIAFLOWS RESTful programming interface to export project data (including images and object annotations) from the source instance to the destination instance. Corresponding code and documentation is available here and can be adapted for specific purposes:
https://github.com/Neubias-WG5/Cytomine-project-migrator.

Manual Import of annotations

In order to be able to compute metrics for benchmarking (optional), ground-truth annotations should also be provided for all uploaded images and encoded with format specified in Section 4 **Problem class, ground truth annotations and reported metrics**. Workflow results use the same formats as ground truth annotations, but to be visualized in BIAFLOWS they are internally converted to polygons and points. It is possible to manually convert annotations from an annotation image mask (or CSV files) to this format with the Python library: https://github.com/Neubias-WG5/biaflows-utilities/tree/master/biaflows/helpers/data_upload.py.
Supported formats include 2D, 3D/2D+t and 3D+t objects. These annotations can then be uploaded to BIAFLOWS and displayed using overlays in the web image viewer as any annotation automatically created by a workflow.

RESTful API documentation

All interactions with BIAFLOWS are performed through a RESTful API. This API enables communication between a BIAFLOWS instance and a client. All the services provided by the API are summarized in a user-friendly way on a website automatically installed with BIAFLOWS installation procedure. From this interface, the documentation can be browsed and API queries / replies directly tested in a playground area:

BIAFLOWS RESTful API documentation can be found here:
https://biaflows.neubias.org/restApiDoc/?doc_url=https://biaflows.neubias.org/restApiDoc/api#

and here from a local instance of BIAFLOWS:
http://biaflows/restApiDoc/?doc_url=http://biaflows/restApiDoc/api

Executing a BIAFLOWS workflow without BIAFLOWS server

It is possible to run a workflow image independently of any BIAFLOWS server. This can for instance be useful to process a local folder of images. For this, first install Docker on the target workstation, then:

- Get the docker image of the workflow from DockerHub **(recommended)**:
  docker pull {remote_image}

  Or, alternatively, build workflow Docker image from source (GitHub repository)
  Inside repository folder: docker build -t {local_image} .

- Prepare an empty folder {DATA_PATH} with a subfolder **/data** and subfolders:
  - **{DATA_PATH}/data/in**: add input images to this folder*****
  - **{DATA_PATH}/data/out**: workflow results are exported to this folder
  - **{DATA_PATH}/data/gt**: leave empty

***** Images should be 8/16-bit TIFF (2D) or 8/16-bit single file OME-TIFF (C,Z,T).
The string **_lbl** is forbidden in image name since it is used to identify ground truth annotation images.

- Run the workflow with the local flag:

```
docker run -v {DATA_PATH}/data:/data -it {image_name} {WORKFLOW_PARAMETERS} --infolder /data/in --gtfolder /data/gt --outfolder /data/out --local
```

This whole procedure is illustrated in the following Python Jupyter notebook:
https://github.com/Neubias-WG5/biaflows_jupyter_local

Notes:

--local (-l): do not download nor upload any content from / to BIAFLOWS. The images (input and ground truth) are read from specified folders. Metrics are optionally displayed to standard output.

For a more fine-grained control over BIAFLOWS interactions:
--no_download (-nd): images and ground truth are not downloaded from BIAFLOWS
--no_annotations_upload (-nau): annotations are not uploaded to BIAFLOWS
--no_metrics_computation (-nmc): metrics are not computed
--no_metrics_upload (-nmu): metrics are not uploaded to BIAFLOWS.

Section 6. Benchmark Metrics

In this Section we describe the benchmark metrics computed by BIAFLOWS. The source code is available from our metrics library: https://github.com/Neubias-WG5/neubiaswg5-utilities/tree/master/neubiaswg5/metrics.

Object Segmentation (ObjSeg)

DICE (DICE)

DICE coefficient is computed as:

2 * AreaOverlap(***X***, ***Y***) / (**Area(** ***X***) + **Area(** ***Y***))

where ***X*** is ground truth binary mask and ***Y*** is prediction binary mask. Object pixels are nonnull pixels in the original masks.

DICE coefficient ranges from 0 (no overlap between segmented objects) to 1 (perfect overlap).

AVERAGE HAUSDORFF DISTANCE (AHD)

The Average Hausdorff Distance (AHD) is:

$$(\mathrm{Avg}(D_1) + \mathrm{Avg}(D_2)) / 2$$

where, for every object pixel of the ground truth mask the minimum distance D_1 to the closest object pixel of the prediction mask is computed (and vice versa, leading to minimum distance D_2 for every object pixel of the prediction mask). Object pixels are all nonnull pixels of the masks, and averages are computed over the object pixels of the respective masks. Lower AHD implies better segmentation and the metric is equal to 0 only for perfect segmentation.

FRACTION OVERLAP

Every object ***R*** from the ground truth label mask is associated to the object ***P*** from the prediction label mask with maximum overlap. The score for this object is computed as:

S = **AreaOverlap** (***R***, ***P***) **/ max**(**Area** (***R***), **Area** (***P***))

The reported metric is the average score ***S*** over all objects ***R***.

A 0.5 fraction overlap can be interpreted as "on average the area of a predicted object overlapping with the ground truth object with largest overlap is half the area of the larger of these two objects". The metric is equal to 1 only for perfect segmentation. This would for instance happen if an object is erroneously split into two objects of the same size, or if two objects of the same size are erroneously merged.

Mean Average Precision

Mean average precision as used in Data Science Bowl 2018 evaluation: https://www.kaggle.com/c/data-science-bowl-2018/overview/evaluation

Intersection over union (**IoU**) between two sets of pixels is calculated as:
IoU(A,B) = (A ∩ B) / (A ∪ B).

The metric calculates IoU between all predicted objects and ground-truth objects. Then IoU for each predicted object is tested with 10 thresholds (0.5, 0.55, ..., 0.95) and if the IoU is greater than the tested threshold, the object is set as true positive (**TP**) for that threshold.

Precision at each threshold value is calculated as
P = TP / (TP + FP + FN),
where **FP** = number of predicted objects - TP and **FN** = number of ground-truth objects - TP.

Average precision (**AP**) of a single image is the mean of precision with 10 different thresholds.

Mean average precision (**mAP**) is the mean of AP for all images.

Spot / object counting (ObjCnt)

RELATIVE_ERROR_COUNT (REC)

The relative error count is computed as the absolute difference between the number of ground truth and prediction objects, normalized to the number of ground truth objects. A perfect count leads to REC = 0.

Spot / object detection (ObjCnt)

CONFUSION_MATRIX (TP, FN, FP)
F1_SCORE (F1)
PRECISION (PR)
RECALL (RE)
Distance RMSE (RMSE)

The set of ground truth and prediction detections are first associated by solving a distance-constrained assignment problem with the Hungarian algorithm. This procedure pairs detections up to a maximum (gating) distance by minimizing their overall distance. Paired detections are considered True Positives (**TP**). In a classification setup, given a classification problem with C distinct classes, a confusion matrix is a square matrix of dimensions C x C where the element a_{ij} ($i \in [0, C[$, $j \in [0, C[$) is the number of samples of class j that are predicted to be class i. For binary classification (C = 2), a_{10} is the number of False Positives (**FP**) and a_{01} is the number of False Negatives (**FN**).

The precision is defined as (see definitions of True Positive and False Positive metrics):

PR = TP / (TP + FP)

The precision reflects the ability of a classifier not to label as positive a sample that is negative. It ranges from 0 (all positive samples are misclassified) to 1 (no false positive). For Multiclass classification ($C > 2$), we use the weighted averaged precision: precision is computed for each class separately and then the resulting precisions are averaged weighted by support (number of positive samples for each class). This weighting strategy accounts for class imbalance.

The Recall is defined as (see definitions of True Positive and False Negative metrics):

RE = TP / (TP + FN)

Intuitively, the recall is the ability of the classifier to find all positive samples. It ranges from 0 (all positive samples misclassified) to 1 (no false negative). For Multiclass classification, we use the same approach as for Precision (**PR**): a support weighted average recall.

The accuracy (ACC) is the proportion of correctly predicted samples (the sum of the diagonal elements of the confusion matrix divided by the total number of samples). The accuracy ranges from 0 (all samples misclassified) to 1 (all samples correctly classified). A classifier that would pick a class at random typically yields accuracy around 1 / **C**. For binary classification, we have:

ACC = (TP + TN) / (TP + TN + FP + FN)

The F1-score is defined as:

F1 = 2 * (PR * RE) / (PR + RE)

where **PR** is the Precision and **RE** is the Recall (see the definition of these metrics). Recall and precision must be analyzed jointly and it makes no sense to benchmark one or the other independently. For instance, for a balanced binary classification problem, it is easy to get a perfect recall of 1 with a constant classifier that would always predict the positive class but this classifier would yield a precision of 0.5 (which is the score a random classifier would get). This is what F1-score attempts to capture. It ranges from 0 (precision and / or recall equal 0) to 1 (all samples correctly classified).

Localization accuracy (root mean square distance) of paired detections between ground truth and predictions. The default gating distance (maximum pairing distance) is set to 5 pixels. For instance, a RMSE distance of 3 means that on average the detected particles (objects) are 3 pixel away from the ground truth particles (objects).

Pixel/Voxel Classification (PixCla)

F1_SCORE (F1)
ACCURACY (ACC)
PRECISION (PR)
RECALL (RE)

See definitions from Spot / object detection (ObjCnt)

Landmark Detection (LndDet)

Number of ground truth landmarks (NREF)
Number of predicted landmarks (NPRED)
Mean distance (MRE): Mean distance from predicted to ground truth landmarks (pix)

Particle Tracking (PrtTrk)

14 metrics from PTC challenge (Supplemental note 3), of which 5 metrics are derived:

1. $\alpha(X, Y) = 1-d(X, Y)/d(X, \emptyset)$. $\emptyset$ denotes a set of dummy tracks; hence, $d(X, \emptyset)$ is the maximum possible total distance (error) from the ground truth. The measure ranges from 0 (worst) to 1 (best), indicating the overall degree of matching of ground truth and predicted tracks without taking into account spurious (non-paired estimated) tracks.

2. $\beta(X, Y) = (d(X, \emptyset)-d(X, Y))/(d(X, \emptyset) + d(Y, \emptyset))$. Y denotes the set of spurious tracks, and $d(Y, \emptyset)$ is the corresponding penalty term. The measure ranges from 0 (worst) to α (best) and is essentially α with a penalization of non-paired estimated tracks.

3. $JSC = TP/(TP + FN + FP)$. This is the Jaccard similarity coefficient for track points. It ranges from 0 (worst) to 1 (best) and characterizes overall particle detection performance. TP (true positives) denotes the number of matching points in the optimally paired tracks; FN (false negatives), the number of dummy points in the optimally paired tracks; and FP (false positives), the number of non-matching points including those of the spurious tracks.

4. $JSC\theta = TP\theta/(TP\theta + FN\theta + FP\theta)$. This is the Jaccard similarity coefficient for entire tracks instead of single track points. Similarly to JSC, it ranges from 0 (worst) to 1 (best). $TP\theta$ denotes the number of predicted tracks paired with ground-truth tracks; $FN\theta$, the number of dummy tracks paired with ground-truth tracks; and $FP\theta$, the number of spurious tracks.

5. RMSE, the r.m.s. error, indicates the overall localization accuracy of matching points in the optimally paired tracks (the TP as in JSC), being a nonnegative number with the upper bound given by the maximum distance specified.

Note: gating distance to pair particles from ground truth and prediction default to 5 pixels.

Object Tracking (ObjTrk)

The metrics are computed from Cell Tracking Challenge: http://celltrackingchallenge.net/ code and are described in detail in: https://doi.org/10.1038/nmeth.4473.

Segmentation accuracy measure (SEG) evaluates the average amount of overlap, being expressed by the Jaccard similarity index, between the ground truth segmentation ground truth and the prediction. Tracking accuracy measure (TRA) is a normalized weighted distance between the prediction and the ground truth, with weights chosen to reflect the effort it takes a human curator to carry out the edits manually (see https://doi.org/10.1371/journal.pone.0144959 for details).

Both SEG and TRA take values in the interval [0, 1], with higher values corresponding to better performance.

Filament Networks Tracing (LooTrc)

NetMets metrics

Geometric False Negative rate (G_{FNR})
Geometric False Positive rate (G_{FPR})
Unmatched voxel rate (UVR)

Citations: Mayerich, D., Bjornsson C.,Taylor J., Roysam B. (2012). NetMets: software for quantifying and visualizing errors in biological network segmentation. BMC Bioinformatics, 13(Suppl 8): S7.

NetMets relies on mapping nodes between ground truth and prediction filament networks. Given two networks $\boldsymbol{N_1}$ and $\boldsymbol{N_2}$, it estimates the integrated length of sections of $\boldsymbol{N_1}$ without correspondence in $\boldsymbol{N_2}$ normalized to $\boldsymbol{N_1}$ length. This is estimated by surrounding $\boldsymbol{N_2}$ by a penalty weighting Gaussian envelope (penalty increase with distance) and integrating along $\boldsymbol{N_1}$. In order to estimate both missed and erroneously detected sections, a bi-directional measurement is performed by inverting the role of the networks: this leads to G_{FNR} and G_{FPR}.

Overall, the more distant nodes are from the nearest node in the other network, the lower the metrics (1: perfect match).

Note: The skeleton masks are first converted to SWC models by sampling them (default sampling step is 3 voxels and default Z ratio is 1).

Unmatched voxel rate (UVR)

Ground truth and prediction skeleton masks are both dilated by a gating distance (default: 5 voxels). The number of object voxels from prediction mask not falling within the gating distance of an object voxel of the ground truth are counted. The same is performed for ground truth object voxels (respect to prediction object voxels). Unmatched Voxel Rate (UVR) is computed as the number of unmatched voxels (two-ways) divided by the sum of the number of object voxels in both skeleton mask. It equals to 0 for perfect matching (within the gating distance) and to 1 for the worse possible matching.

Filament Tree Tracing (TreTrc)

Geometric False Negative rate (G_{FNR})
Geometric False Positive rate (G_{FPR})

See metrics definition from Filament Networks Tracing (LooTrc). The note does not apply since workflows directly outputs the trees in SWC format.

Supplemental References

1. Lehmussola, A., Ruusuvuori, P., Selinummi, J., Huttunen, H., Yli-Harja, O. (2007). Computational framework for simulating fluorescence microscope images with cell populations. IEEE Transactions on Medical Imaging. 26(7), 1010–1016.

2. Caicedo, J.C., Goodman, A., Karhohs, K.W., Cimini, B.A., Ackerman, J., Haghighi, M., Heng, C., Becker, T., Doan, M., McQuin, C., et al. (2019). Nucleus segmentation across imaging experiments: the 2018 Data Science Bowl. Nature Methods 16, 1247–1253.

3. He, K., Gkioxari, G., Dollár, P., Girshick., R. (2017). Mask R-CNN. Proceedings of IEEE International Conference on Computer Vision (ICCV), 2980–2988.

4. Hollandi, R., Szkalisity, A., Toth, T., Tasnadi, E., Molnar, C., Mathe, B., Grexa, I., Molnar, J., Balind, A., Gorbe, M., et al. (2019). A deep learning framework for nucleus segmentation using style transfer. bioRxiv: 580605.

5. Ronneberger, O., Fischer, P., Brox., T. (2015). U-Net: Convolutional networks for biomedical image segmentation. Proceedings of Medical Image Computing and Computer-Assisted Intervention (MICCAI), Springer, Lecture Notes in Computer Science 9351, 234–241.

6. Van Valen, D.A., Kudo, T., Lane, K.M, Macklin, D.N., Quach, N.T., DeFelice, M.M., Maayan, I., Tanouchi, Y., Ashley, E.A., Covert, M.W. (2016). Deep learning automates the quantitative analysis of individual cells in live-cell imaging experiments. PLOS Computational Biology 12(11), e1005177.
